# Trk-fused gene plays a critical role in diet-induced adipose tissue expansion and is also involved in thyroid hormone action

**DOI:** 10.1093/pnasnexus/pgae150

**Published:** 2024-04-09

**Authors:** Takeshi Yamamotoya, Yukino Ohata, Yasuyuki Akasaka, Shun Hasei, Masa-Ki Inoue, Yusuke Nakatsu, Machi Kanna, Hiroki Yamazaki, Akifumi Kushiyama, Midori Fujishiro, Hiraku Ono, Hideyuki Sakoda, Tetsuya Yamada, Hisamitsu Ishihara, Tomoichiro Asano

**Affiliations:** Department of Biomedical Chemistry, Graduate School of Biomedical and Health Sciences, Hiroshima University, 1-2-3 Kasumi, Minami-ku, Hiroshima City, Hiroshima 734-8553, Japan; Department of Biomedical Chemistry, Graduate School of Biomedical and Health Sciences, Hiroshima University, 1-2-3 Kasumi, Minami-ku, Hiroshima City, Hiroshima 734-8553, Japan; Department of Biomedical Chemistry, Graduate School of Biomedical and Health Sciences, Hiroshima University, 1-2-3 Kasumi, Minami-ku, Hiroshima City, Hiroshima 734-8553, Japan; Department of Biomedical Chemistry, Graduate School of Biomedical and Health Sciences, Hiroshima University, 1-2-3 Kasumi, Minami-ku, Hiroshima City, Hiroshima 734-8553, Japan; Department of Biomedical Chemistry, Graduate School of Biomedical and Health Sciences, Hiroshima University, 1-2-3 Kasumi, Minami-ku, Hiroshima City, Hiroshima 734-8553, Japan; Department of Biomedical Chemistry, Graduate School of Biomedical and Health Sciences, Hiroshima University, 1-2-3 Kasumi, Minami-ku, Hiroshima City, Hiroshima 734-8553, Japan; Department of Biomedical Chemistry, Graduate School of Biomedical and Health Sciences, Hiroshima University, 1-2-3 Kasumi, Minami-ku, Hiroshima City, Hiroshima 734-8553, Japan; Department of Internal Medicine, Teikyo University School of Medicine, 2-11-1 Kaga, Itabashi-ku, Tokyo 173-8606, Japan; Department of Pharmacotherapy, Meiji Pharmaceutical University, 2-522-1 Noshio, Kiyose City, Tokyo 204-8588, Japan; Division of Diabetes and Metabolic Diseases, Nihon University School of Medicine, 30-1 Oyaguchikamicho, Itabashi-ku, Tokyo 173-8610, Japan; Department of Clinical Cell Biology, Graduate School of Medicine, Chiba University, 1-8-1 Inohana, Chuo-ku, Chiba City, Chiba 260-8670, Japan; Department of Bioregulatory Sciences, Faculty of Medicine, University of Miyazaki, Miyazaki 889-1692, Japan; Department of Molecular Endocrinology and Metabolism, Tokyo Medical and Dental University, Yushima 1-5-45, Bunkyo-ku, Tokyo 113-8510, Japan; Division of Diabetes and Metabolic Diseases, Nihon University School of Medicine, 30-1 Oyaguchikamicho, Itabashi-ku, Tokyo 173-8610, Japan; Department of Biomedical Chemistry, Graduate School of Biomedical and Health Sciences, Hiroshima University, 1-2-3 Kasumi, Minami-ku, Hiroshima City, Hiroshima 734-8553, Japan

**Keywords:** Trk-fused gene (TFG), adipogenesis, adipose expansion, thyroid hormone, carbohydrate responsive element binding protein (ChREBP)

## Abstract

Mutations in the Trk-fused gene (TFG) cause hereditary motor and sensory neuropathy with proximal dominant involvement, which reportedly has high co-incidences with diabetes and dyslipidemia, suggesting critical roles of the TFG in metabolism as well. We found that TFG expression levels in white adipose tissues (WATs) were elevated in both genetically and diet-induced obese mice and that TFG deletion in preadipocytes from the stromal vascular fraction (SVF) markedly inhibited adipogenesis. To investigate its role in vivo, we generated tamoxifen-inducible adipocyte-specific TFG knockout (AiTFG KO) mice. While a marked down-regulation of the peroxisome proliferator-activated receptor gamma target, de novo lipogenesis (DNL), and mitochondria-related gene expressions were observed in subcutaneous WAT (scWAT) from AiTFG KO mice, these effects were blunted in SVF-derived adipocytes when the TFG was deleted after differentiation into adipocytes, implying cell nonautonomous effects. Intriguingly, expressions of thyroid hormone receptors, as well as carbohydrate responsive element-binding protein β, which mediates the metabolic actions of thyroid hormone, were drastically down-regulated in scWAT from AiTFG KO mice. Reduced DNL and thermogenic gene expressions in AiTFG KO mice might be attributable to impaired thyroid hormone action in vivo. Finally, when adipocyte TFG was deleted in either the early or the late phase of high-fat diet feeding, the former brought about an impaired expansion of epididymal WAT, whereas the latter caused prominent adipocyte cell death. TFG deletion in adipocytes markedly exacerbated hepatic steatosis in both experimental settings. Collectively, these observations indicate that the TFG plays essential roles in maintaining normal adipocyte functions, including an enlargement of adipose tissue, thyroid hormone function, and thermogenic gene expressions, and in preserving hypertrophic adipocytes.

**Significance Statement**

Adipose tissues expand in response to excessive caloric intake by increasing either the number (hyperplasia) or size (hypertrophy) of adipocytes. The Trk-fused gene (TFG) was shown to be a novel regulator of adipogenesis, and its deletion in the early phase of high-fat diet feeding markedly impaired hyperplastic expansion of epididymal white adipose tissue (WAT), whereas its deletion in hypertrophic adipocytes caused massive adipocyte death, both of which exacerbated hepatic steatosis. Furthermore, we found that the TFG regulates responsiveness to thyroid hormone in vivo, which modulates de novo lipogenesis and thermogenic gene expressions in subcutaneous WAT. Our findings highlight essential roles of the TFG in enlargement and many adipose tissue functions, with possible involvement in generating ectopic lipid accumulation.

## Introduction

Obesity, a major health concern worldwide, is associated with insulin resistance and type 2 diabetes mellitus. However, obesity is not necessarily a prerequisite for the development of this form of diabetes, since even lean individuals can develop type 2 diabetes depending on their genetic backgrounds, as is especially commonly observed in Asian populations (1). The ability of adipose tissue to adequately store excessive caloric intake as fat is crucial to maintaining a metabolically healthy state, and lipid spillover into extra-adipose tissues such as the liver and muscles, collectively known as ectopic lipid accumulation, causes insulin resistance and predisposes the individual to developing type 2 diabetes. Indeed, severe insulin resistance and ectopic lipid accumulation are the major characteristics of patients with both lipodystrophy (2) and various murine models of lipodystrophy such as transgenic mice overexpressing sterol regulatory element-binding protein-1c in adipose tissue under the aP2-promotor (3), fat-specific nuclear receptor peroxisome proliferator-activated receptor gamma (PPARγ) knockout (KO) mice (4), and adipocyte-specific tamoxifen-inducible insulin and insulin-like growth factor-1 (IGF-1) receptor KO mice (5). Furthermore, there are normal-weight subjects who are metabolically unhealthy, with elevated risks of all-cause mortality and cardiovascular events (6–8). Low subcutaneous leg fat mass, in addition to impaired insulin secretion capacity and insulin resistance, was reported to be prevalent risk factors for this metabolically unhealthy state in normal-weight individuals (7, 9, 10), supporting the significant role of adequate adipose expansion capacity in maintaining a metabolically healthy state.

Adipose tissue expansion requires an increase in the size (hypertrophy) and/or number (hyperplasia) of adipocytes, the latter achieved by the proliferation of adipocyte precursor cells and their subsequent differentiation into mature adipocytes (11). In humans, despite the number of adipocytes reportedly being determined during childhood and adolescence and remaining stable in adulthood (12), the fact that ∼10% of adipocytes are renewed annually even in adults (12) and the observation that fat cell numbers increase in adults after 8 weeks of overfeeding (13) suggest that even in adults, adipose tissue expansion is apparently regulated not only by adipocyte hypertrophy but also by adipocyte hyperplasia.

The Trk-fused gene (TFG) was initially recognized as an oncogene, the N-terminal half of which was fused with neurotrophic tyrosine kinase receptor 1 (also known as TrkA) (14). The functions of the TFG itself remained unknown for some time, but recent findings have suggested its essential role in coat protein complex II (COPII)-mediated vesicle transport (15–17) and its clinical significance as a causative gene for several neurodegenerative disorders such as hereditary motor and sensory neuropathy with proximal dominant involvement (HMSN-P) (18). Clinically, HMSN-P shows high associations with both diabetes and dyslipidemia (19). Surprisingly, we found TFG expression to be significantly up-regulated in adipose tissues from obese mice, which prompted us to investigate the roles of the TFG in adipogenesis and its potential contribution(s) to obesity development. To gain insights into the roles of adipocyte TFG in vivo, we generated tamoxifen-inducible adipocyte-specific TFG KO (AiTFG KO) mice and examined the metabolic effects produced by high-fat diet (HFD) feeding in two contexts, specifically, the deletion of adipocyte TFG in either the early or the late phase of HFD feeding. To our knowledge, this is the first study to demonstrate the significance of the TFG in adipogenesis and adipose expansion as well as its necessity for the survival of enlarged adipocytes.

## Materials and methods

### Animals

AiTFG KO mice were generated by crossing TFG-floxed mice (20) with Adipoq-CreERT2 mice (purchased from the Jackson Laboratory; stock no. 025124) and then intraperitoneally injecting tamoxifen (Sigma-Aldrich, Darmstadt, Germany) dissolved in corn oil into 8-week-old (unless otherwise noted) male mice at a dose of 50 mg/kg for 5 consecutive days, according to a previous report (21). As controls, we prepared TFG-floxed mice similarly injected with tamoxifen. C57BL6/J, AKR, A/J, BALB/c, and ob/ob mice were purchased from Japan SLC, Inc. (Hamamatsu, Japan). All mice were housed in temperature- and light-controlled rooms with free access to food and water. The HFD was purchased from Research Diets (New Brunswick, NJ, USA; D12492). CL-316,243 (1 mg/kg; Abcam, Cambridge, UK) was administered by intraperitoneal injection, and analyses were conducted 24 h later unless otherwise noted. To render the mice hypothyroid, 0.1% 2-mercapto-1-methylimidazole (MMI; Sigma-Aldrich) and 1% NaClO_4_ (Wako, Osaka, Japan) dissolved in drinking water were administered for 4 weeks (22). Half of the mice were injected with 0.25 mg/kg 3,3′,5-triiodo-L-thyronine (T3; Sigma-Aldrich) intraperitoneally for the final 5 days. All animals were handled in accordance with the Guidelines for the Care and Use of Experimental Animals published by Hiroshima University, and all protocols were approved by the Institutional Review Board of Hiroshima University.

### Metabolic studies

Whole venous blood was obtained from the tail veins of the mice, and their glucose levels were measured using Medisafe Fit Smile (Terumo, Tokyo, Japan). Liver triglycerides (TGs) were extracted by homogenizing 50 mg of liver tissue in 1.25 mL of chloroform/methanol (chloroform:methanol = 2:1 (v/v)). After adding 0.4 mL of chloroform and 0.4 mL of water, the samples were vortexed and centrifuged for 3 min at 3,000 rpm. The lower layer was collected, evaporated, and dissolved with 10% Triton X-100 in isopropanol. TG concentrations were measured using the TG E-test (Wako).

### Isolation of primary adipocytes and stromal vascular fraction, and differentiation of stromal vascular fraction cells into mature adipocytes

Primary adipocytes and stromal vascular fraction (SVF) were isolated according to a previous report (23). Briefly, freshly excised mouse adipose depots were minced and then digested with 0.8 mg/mL collagenase type 2 (Worthington, Lakewood, NJ, USA) for 75 min in a shaking water bath, kept at 37 °C, and filtered through a 200 μm filter to remove debris. After centrifugation, floating adipocytes were collected to serve as primary adipocytes. The supernatant was removed, and the SVF pellet was resuspended in Dulbecco's modified Eagle's medium containing 10% fetal bovine serum and filtered through 70 and 40 μm filters. SVF cells were seeded into a 24-well culture dish, and 2 days after reaching confluence, differentiated into adipocytes by switching to an induction medium containing 0.2 μM insulin, 0.25 μM dexamethasone, 0.5 mM isobutylmethylxanthine, and 1 μM rosiglitazone (day 0). From day 2, the medium was switched to Dulbecco's modified Eagle's medium containing 10% fetal bovine serum and 0.2 μM insulin every other day.

### Adeno-associated virus vector

Adeno-associated virus (AAV)-EGFP and AAV-Cre vectors were produced using the AAVpro Helper Free System (Takara, Kusatsu, Japan) in combination with the pAAV-DJ vector purchased from Cell Biolabs, Inc. (San Diego, CA, USA). Viral titers were determined using an AAVpro Titration Kit (for real-time PCR) Ver.2 (Takara), and SVF cells or SVF-derived adipocytes were infected with AAV vectors at a multiplicity of infection of 2,000.

### Western blotting

Tissue samples were homogenized with a lysis buffer containing 50 mM Tris-HCl (pH 7.4), 150 mM NaCl, 1 mM ethylenediamine tetraacetic acid, 1% Triton X-100, 1 mM NaF, 1 mM Na_3_VO_4_, and protease inhibitor cocktail (cOmplete; Roche, Basel, Switzerland), and then immunoblotting was performed as previously reported (24). Antibodies were purchased from Abcam (TFG [ab156866] and uncoupling protein 1 (UCP1) [ab23841]), Santa Cruz Biotechnology (Dallas, TX, USA; β-actin [sc-47778] and PPARγ [sc-7273]), Proteintech (Rosemont, IL, USA; glyceraldehyde-3-phosphate dehydrogenase (GAPDH) [60004-1-Ig] and Lamin B1 [66095-1-Ig]), and Cell Signaling Technology (Danvers, MA, USA; CCAAT enhancer binding protein alpha (C/EBPα) [#8178], C/EBPβ [#3087], C/EBPδ [#2318], PPARγ [#2435], perilipin 1 (Plin1) [#9349], adiponectin (Adipoq) [#2789], Akt [#4691], phospho-Akt (Ser473) [#9271], phospho-protein kinase A (PKA) substrate [#9624], phospho-hormone-sensitive lipase (HSL) (Ser563) [#4139], phospho-HSL (Ser660) [#45804], HSL [#18381]).

### Quantitative real-time PCR

Total RNA was isolated and reverse-transcribed to obtain cDNA, as reported previously (24). Real-time PCR was carried out using a CFX Opus 96 real-time PCR system (Bio-Rad, Hercules, CA, USA) with Brilliant III Ultra-Fast SYBR Green QPCR Master Mix (Agilent Technologies, Santa Clara, CA, USA). The sequences of the primers used in these experiments are listed in Table S2.

### Histological analysis

Tissues were excised and immediately fixed with 4% formaldehyde in phosphate-buffered saline (PBS) overnight and embedded in paraffin. Sections were cut and stained with hematoxylin and eosin. Immunostaining was performed, as previously described (24), using the respective primary antibodies against Plin1 (Cell Signaling Technology, #9349), F4/80 (Cell Signaling Technology, #70076), and UCP1 (Abcam, ab23841).

Oil Red O staining in SVF-derived adipocytes was performed as follows. Cells were washed twice with PBS, fixed with 4% formaldehyde for 10 min, and washed again with PBS twice. After immersion in 60% isopropanol for 1 min, Oil Red O (Sigma-Aldrich), which had been dissolved in 60% isopropanol and filtered through a 0.45-μm pore size membrane, was added to the cells for 15 min. The cells were then washed with PBS three times and finally subjected to microscopic observation.

### Statistical analysis

Statistical analyses were performed employing EZR (Saitama Medical Center, Jichi Medical University, Saitama, Japan) (25). Values are presented as mean ± SE. We used Student's unpaired t test for comparisons between two groups, and ANOVA followed by the post hoc Tukey's test or Dunnett's test for multiple comparisons. We considered *P* < 0.05 to be indicative of a statistically significant difference.

## Results

### TFG expression levels are increased in white adipose tissue from ob/ob mice and HFD-fed mice

**Fig. 1.**
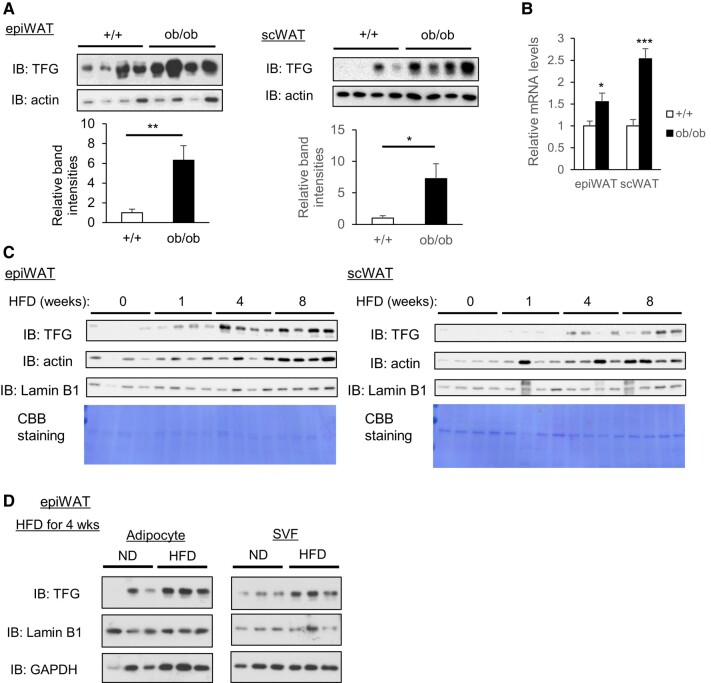
TFG expression is increased in WAT from obese mice. A, B) TFG protein levels (A) and mRNA levels (B) in WAT from wild-type (+/+) and ob/ob mice (*n* = 8). C) TFG protein levels in epiWAT (left panel) and scWAT (right panel) in C57BL6/J male mice fed HFD for 0, 1, 4, and 8 weeks. Lamin B1 as a nuclear marker, Coomassie Brilliant Blue (CBB) staining as a reference for total protein abundance. D) TFG protein levels in isolated primary adipocytes (left panel) and SVF (right panel) in epiWAT from C57BL6/L male mice fed ND or HFD for 4 weeks. *P < 0.05, **P < 0.01, ***P < 0.001.

First, we examined whether TFG expression levels are altered in the adipose tissues of mice with genetic or diet-induced obesity. The TFG protein and mRNA levels were dramatically increased in both epididymal white adipose tissue (epiWAT) and inguinal subcutaneous adipose tissue (scWAT) from ob/ob mice, when compared with those from wild-type mice (Fig. 1A and B). HFD feeding of wild-type mice also increased adipose TFG expression, especially in epiWAT, as well as in scWAT, although to a lesser extent (Fig. 1C). Fractionation of adipose tissue into adipocytes and the SVF revealed that the TFG increase in epiWAT in response to HFD feeding occurred in both adipocytes and SVF (Fig. 1D). Unlike in WAT, TFG expression was decreased in brown adipose tissue (BAT) from ob/ob mice (Fig. S1A) and remained essentially unchanged in response to HFD feeding (Fig. S1B).

The significant role of adipocyte TFG in the development and expansion of adipose tissue was also suggested by the data comparing the mRNA or protein abundance of adipose TFG among four strains of 8-week-old male mice (A/J, BALB/c, C57BL6/J, and AKR) fed the HFD for 1 week. Surprisingly, the levels of TFG protein expression in WAT varied markedly among these mouse strains, being especially abundant in A/J and AKR (Fig. S2A), which had larger WAT volumes than BALB/c and C57BL6/J mice (Fig. S2B). Consistently, TFG protein levels showed significant positive correlations with WAT volumes in both epiWAT and scWAT (Fig. S2C). Interestingly, in addition to body weight and WAT volume, TFG mRNA levels showed a clear positive correlation with body weight gain during the period of HFD feeding only in epiWAT (Fig. S2D).

### The TFG is essential for adipogenesis

Previous experiments have elegantly shown that in male mice, HFD feeding induces the proliferation of preadipocytes and subsequent differentiation into mature adipocytes, specifically in epiWAT, but not in scWAT, i.e. epiWAT undergoes hyperplastic as well as hypertrophic adipose expansion, whereas scWAT undergoes only hypertrophic expansion (26, 27). Based on these observations that HFD feeding resulted in a more pronounced increase in TFG expression in epiWAT than in scWAT and that the TFG protein increase was observed not only in the adipocyte fraction but also in SVF, which includes preadipocytes, we surmised that the TFG might serve essential roles in HFD-induced adipogenesis.

**Fig. 2.**
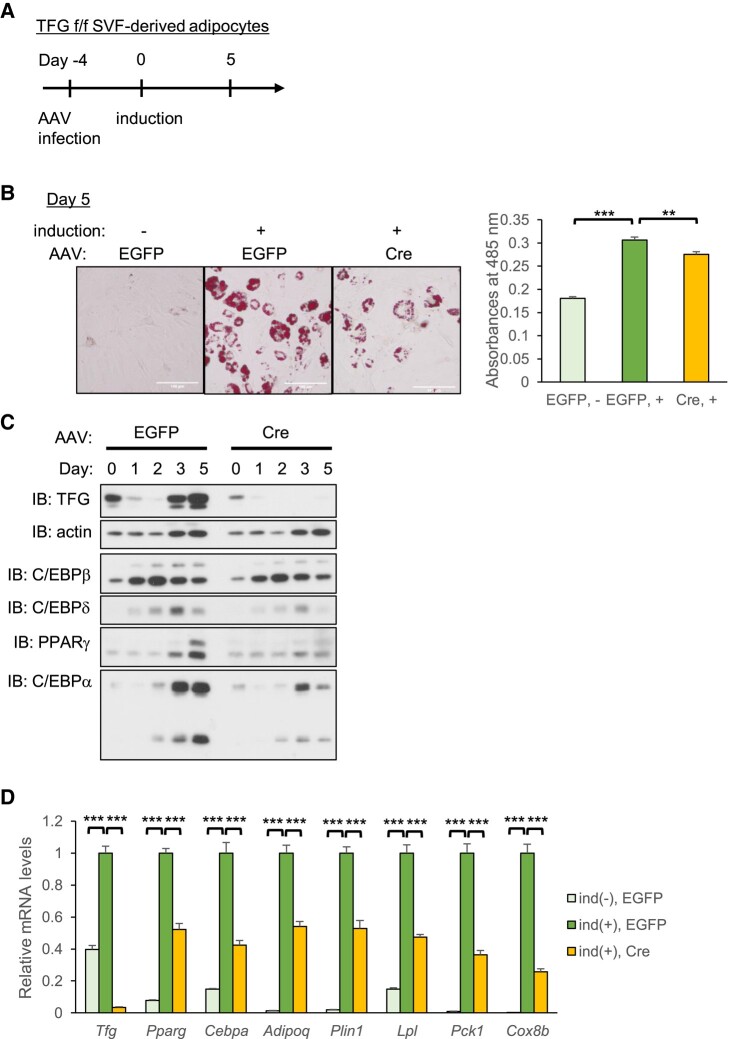
The TFG is essential for adipogenesis. SVF isolated from scWAT of TFG f/f mice were infected with AAV-EGFP (control) or AAV-Cre (day −4) and were then differentiated into mature adipocytes (from days 0 to 5). A) The time course of the experiment. B) Oil Red O staining performed on day 5 (scale bar: 100 μm; left panel). After the staining procedure, intracellular Oil Red O was dissolved out with 100% isopropanol and the absorbances at 485 nm were measured (right panel). C) A Western blot analysis of adipogenic transcription factors on days 0, 1, 2, 3, and 5. D) mRNA levels on day 5 (*n* = 4). ind(−): not switched to the induction medium on day 0. **P < 0.01, ***P < 0.001.

To explore this possibility, SVF cells isolated from scWAT of TFG-floxed (f/f) mice were infected with AAVs expressing either Cre or EGFP as a control and then differentiated into mature adipocytes (Fig. 2A). As expected, adipocyte differentiation was significantly impaired by TFG deletion, as shown by lipid accumulation (Fig. 2B) and adipocyte-specific protein and mRNA levels (Fig. 2C and D). Of note, deleting the TFG after the induction of adipogenesis, i.e. infecting AAV-Cre on day 1, also impaired differentiation into mature adipocytes (Fig. S3A and B), although the effect was rather modest compared with deleting the TFG prior to induction (Fig. 2C and D).

### TFG deletion in mature adipocytes down-regulates PPARγ target, de novo lipogenesis, and mitochondria-related gene expressions, predominantly in scWAT

**Fig. 3.**
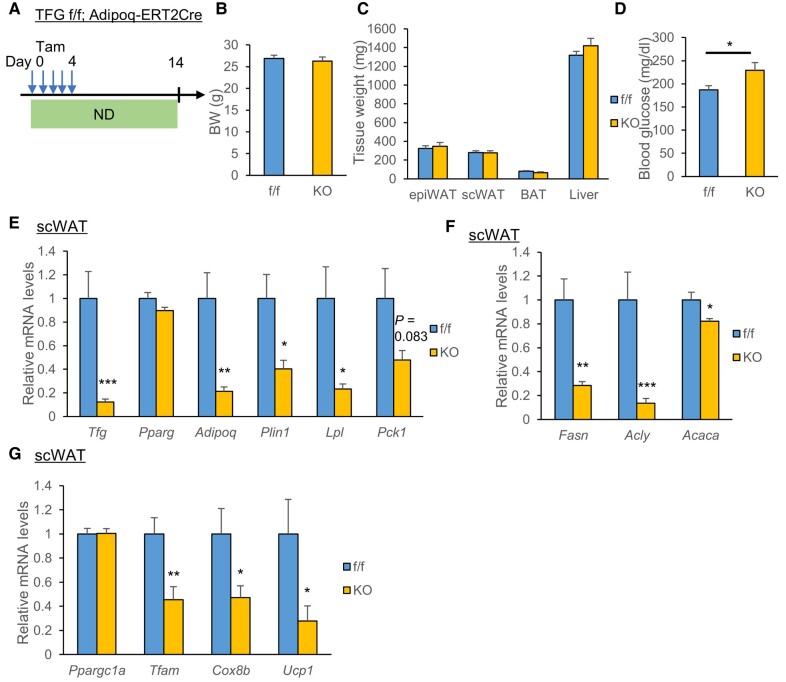
TFG deletion down-regulates the PPARγ target, DNL, and mitochondria-related gene expressions in scWAT. Tamoxifen (50 mg/kg) was intraperitoneally injected into TFG f/f (f/f) and TFG f/f Adipoq-CreERT2 (KO) mice for 5 consecutive days (from day 0 to 4), and analysis was conducted on day 14. A) The time course of the experiment. B) Body weight (*n* = 8–9). C) Tissue weight (*n* = 8–9). D) Blood glucose levels (fed ad libitum) (*n* = 8–9). E–G) mRNA levels of the *Tfg*, *Pparg*, and its target genes (E), DNL genes (F), and genes related to mitochondrial function and thermogenesis (G) in scWAT (*n* = 8). *P < 0.05, **P < 0.01, ***P < 0.001. ND, normal diet.

Next, to examine the functions of the TFG in differentiated mature adipocytes, tamoxifen-inducible AiTFG KO mice were generated by crossing TFG-floxed mice with Adipoq-CreERT2 mice and intraperitoneally injecting tamoxifen for 5 consecutive days (Fig. 3A). Two weeks after the tamoxifen injections, body weights of AiTFG KO mice were similar to those of the controls (Fig. 3B). The volumes of WAT and BAT were similar in the two groups, whereas the hepatic volumes of AiTFG KO mice were slightly larger than those of the control mice (Fig. 3C), and the enlargement was statistically significant when normalized by body weight (Fig. S4A). Ad libitum blood glucose levels were significantly higher in AiTFG KO mice than in the controls (Fig. 3D). Consistently, AiTFG KO mice tended to have higher glucose levels according to intraperitoneal glucose tolerance testing (Fig. S4B) and showed significantly higher glucose levels on the insulin tolerance test (Fig. S4C) and the pyruvate tolerance test (Fig. S4D), both of which suggested enhanced hepatic gluconeogenesis.

To identify the molecular pathways affected by TFG deletion, total RNA extracted from scWAT of either AiTFG KO or control mice was subjected to microarray analysis. Among a total of 65,956 genes analyzed, 266 were up-regulated and 476 were down-regulated by more than 4-fold with statistical significance (*P* < 0.05) in scWAT from AiTFG KO mice when compared with the controls. The pathway analysis results are shown in Table S1. Besides down-regulation of PPAR signaling and fatty acid biosynthesis, the pathways related to mitochondrial function, such as the electron transport chain, tricarboxylic acid (TCA) cycle, and fatty acid oxidation, were significantly down-regulated in scWAT from AiTFG KO mice. Consistently, quantitative analyses using real-time PCR verified the down-regulation of PPARγ target genes (Fig. 3E), genes regulating de novo lipogenesis (DNL; Fig. 3F), and genes related to mitochondrial function and thermogenesis (Fig. 3G) in scWAT from AiTFG KO mice. Down-regulation of these genes was not apparent in epiWAT (Fig. S4E–G, left panels), possibly due to lower efficiency of TFG deletion in epiWAT. Of note, despite efficient deletion of the TFG, these gene expression phenotypes were barely detectable, except for that of the PPARγ target gene *Adipoq*, in BAT (Fig. S4E–G, right panels).

Interestingly, however, when SVF-derived adipocytes isolated and differentiated from scWAT of TFG f/f mice were infected with AAV-Cre on day 4 and gene expressions on day 12 were analyzed (Fig. S5A and B), AAV-Cre infection efficiently deleted the TFG (Fig. S5C) but did not alter the PPARγ target (Fig. S5D), DNL (Fig. S5E), or mitochondria-related gene (Fig. S5F) expressions.

These results suggest that down-regulation of the PPARγ target, DNL, and mitochondria-related gene expressions observed in scWAT upon TFG deletion is not cell-autonomous and presumably require additional factor(s) present only in vivo.

### Induction of *Ucp1* mRNA by adrenergic stimulation is impaired in scWAT from AiTFG KO mice

A recent study demonstrated the cooperation between β3-adrenergic signaling and thyroid hormone in regulating thermogenic and lipogenic gene expressions (28). Therefore, we investigated the response to either adrenergic stimulation or thyroid hormone in AiTFG KO mice, focusing especially on their actions in scWAT.

**Fig. 4.**
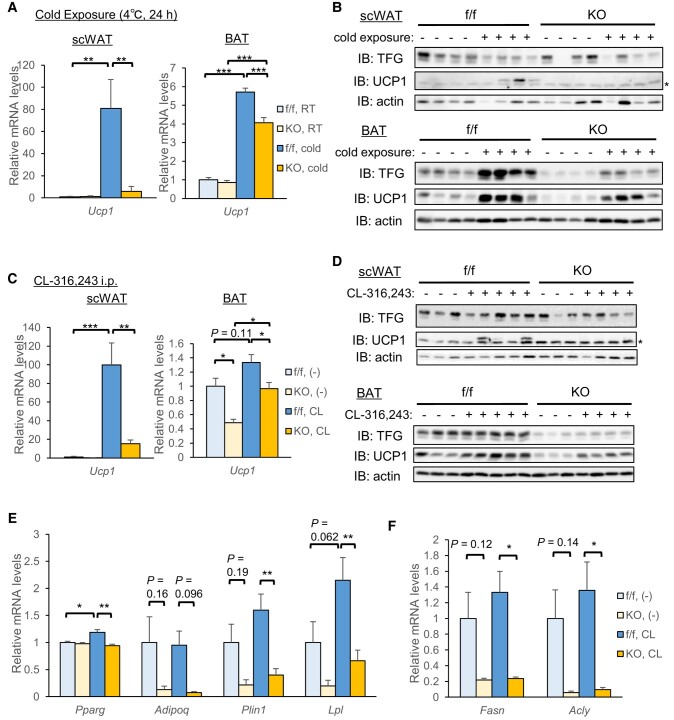
Adrenergic Ucp1 induction is impaired in scWAT from AiTFG KO mice. A) *Ucp1* mRNA levels in scWAT (left panel) and in BAT (right panel) in response to cold exposure (4 °C, 24 h; *n* = 10–12). B) A Western blot analysis in scWAT (upper panel) and in BAT (lower panel). The asterisk (*) indicates a nonspecific band. C–F) Mice were intraperitoneally injected with the β3 agonist CL-316,243 (1 mg/kg), and the analysis was conducted 24 h later. C) mRNA levels of *Ucp1* in scWAT (left panel) and in BAT (right panel) (*n* = 5–7). D) Western blot analysis in scWAT (upper panel) and in BAT (lower panel). The asterisk (*) indicates a nonspecific band. E, F) mRNA levels of *Pparg* and its target genes (E) and DNL genes (F) in scWAT (*n* = 5–7). *P < 0.05, **P < 0.01, ***P < 0.001.

We began by exposing mice to a cold environment (4°C, 24 h) in order to examine gene expressions in response to sympathetic nerve activation. Cold exposure drastically induced *Ucp1* mRNA, which resulted in UCP1 protein being detectable in scWAT from control mice, a response which was significantly blunted in scWAT from AiTFG KO mice (Fig. 4A, left panel and Fig. 4B, upper panel).

Intraperitoneal injection of CL-316,243 (β3 agonist) also induced *Ucp1* mRNA and UCP1 protein in scWAT from control mice, and the diminution was similar in scWAT from AiTFG KO mice (Fig. 4C, left panel and Fig. 4D, upper panel), indicating that impaired Ucp1 induction in scWAT from AiTFG KO mice in response to cold exposure was not attributable to denervation of sympathetic nerve terminals. Histologically, UCP1 immunostaining revealed browning in scWAT from control mice in response to injecting CL-316,243 for 7 consecutive days, which was apparently impaired in scWAT from AiTFG KO mice (Fig. S6A–C). In contrast to *Ucp1* mRNA, PPARγ target genes (Fig. 4E) and DNL genes (Fig. 4F) were down-regulated in scWAT from AiTFG KO mice independently of CL injection, suggesting down-regulation of the PPARγ target and DNL genes in scWAT from AiTFG KO mice to be independent of adrenergic signaling.

Similarly in BAT, *Ucp1* mRNA and UCP1 protein were increased by cold exposure (Fig. 4A, right panel and Fig. 4B, lower panel) and by CL-316,243 injection (Fig. 4C, right panel and Fig. 4D, lower panel), albeit less significantly. Although both expression levels were significantly lower in AiTFG KO mice than in the controls, the difference between the genotypes was less marked than the difference in scWAT.

Of note, there was no difference between the genotypes in mRNA levels of the β3-adrenergic receptors (*Adrb3*) in either scWAT or BAT (Fig. S7A). To evaluate the downstream signaling, we injected mice with CL-316,243, sacrificed them 1 h later and analyzed the phospho-PKA substrate levels as well as the phosphorylations of HSL at Ser563 and Ser660, both of which are well-known phosphorylation targets of PKA. Interestingly, the phosphorylations of these PKA targets upon CL-316,243 injection appeared to be slightly impaired in scWAT from AiTFG KO mice compared with the control (Fig. S7B), whereas there was no difference between the genotypes in BAT (Fig. S7C). These data collectively point to TFG deletion impairing adrenergic signaling and subsequent Ucp1 induction and, furthermore, these responses are essentially specific to scWAT.

### Thyroid hormone action is impaired in scWAT from AiTFG KO mice

Carbohydrate responsive element-binding protein (ChREBP, gene name *Mlxipl*) is a transcription factor, which is activated in response to simple carbohydrates, such as glucose and fructose and regulates the transcription of DNL genes, including *Fasn* and *Acly* (29). ChREBP is abundantly expressed in lipogenic tissues, such as the liver, intestine, and adipose tissue (30). In addition, several recent studies have suggested that ChREBP is induced by thyroid hormone and mediates metabolic actions of thyroid hormone in adipose tissue (31), the liver (32), and pancreatic β-cells (33). The mRNA levels of ChREBP were revealed to be markedly down-regulated in scWAT from AiTFG KO mice (Fig. S8A), without significant impairment of insulin-induced Akt phosphorylation (Fig. S8B) or glucose uptake (Fig. S8C). Furthermore, mRNA levels of thyroid hormone receptors (*Thra* and *Thrb*) were significantly reduced in scWAT from AiTFG KO mice (Fig. S8D). These observations prompted us to speculate that the observed ChREBP down-regulation might reflect impaired thyroid hormone action in scWAT from AiTFG KO mice.

**Fig. 5.**
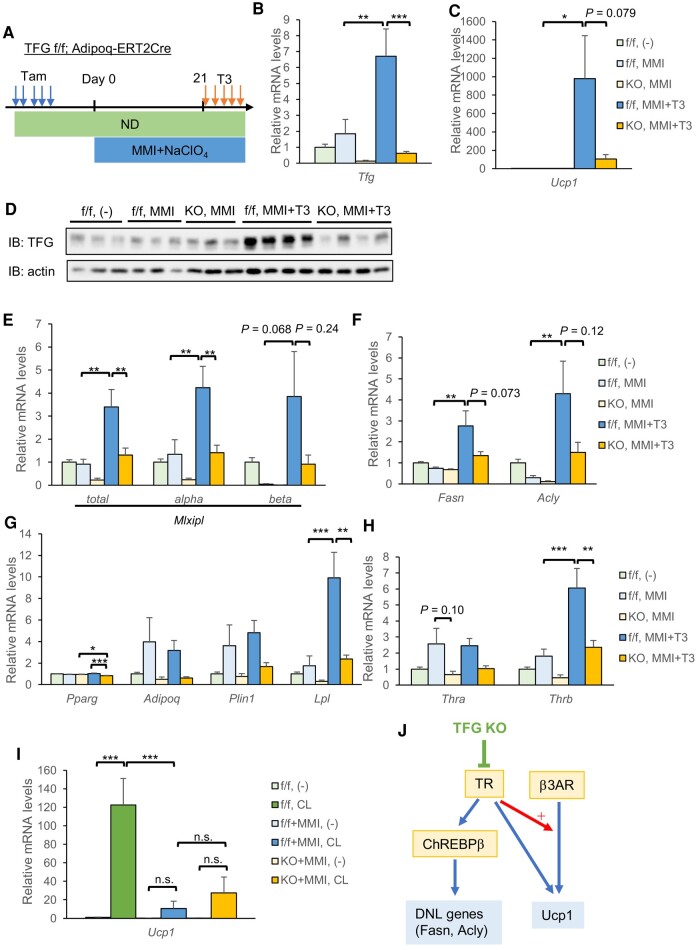
Thyroid hormone action is impaired in scWAT from AiTFG KO mice. A–H) Mice were rendered hypothyroid by administering MMI and NaClO_4_ via drinking water for 4 weeks and then received intraperitoneal injections of T3 (0.25 mg/kg) or vehicle for the final 5 days. A) The time course of the experiment. B, C) mRNA levels of the *Tfg* (B) and *Ucp1* (C) (*n* = 7–9). D) A Western blot analysis. E–H) mRNA levels of ChREBP (*Mlxipl*) (E), DNL genes (F), *Pparg* and its target genes (G), and thyroid hormone receptors (H) in scWAT (*n* = 7–9). I) Mice were rendered hypothyroid by administering MMI and NaClO_4_ and were then injected with CL-316,243 (1 mg/kg) or vehicle 24 h before being sacrificed. *Ucp1* mRNA levels in scWAT (*n* = 4–6). J) Working hypothesis. The thyroid hormone induces ChREBP (including the especially potent form, ChREBPβ) and up-regulates DNL genes. The thyroid hormone also up-regulates Ucp1 either by potentiating adrenergic signaling (28) or by directly inducing its transcription (31). The TFG probably regulates thyroid hormone sensitivity in scWAT and influences the expressions of DNL genes and Ucp1. *P < 0.05, **P < 0.01, ***P < 0.001.

To evaluate the effects of thyroid hormone, we rendered mice hypothyroid by administering MMI along with NaClO_4_ for 4 weeks, with half of the mice being injected with T3 for the final 5 days to induce hyperthyroid status (22, 31) (Fig. 5A).

Notably, T3 injection significantly increased *Tfg* mRNA in scWAT (Fig. 5B), and this induction was also confirmed at protein levels (Fig. 5D). T3 significantly induced UCP1 in scWAT from TFG f/f mice, a response which was apparently blunted in AiTFG KO mice (Fig. 5C). It is noteworthy that, in BAT, unlike in scWAT, T3 injection did not increase *Ucp1* mRNA levels, which is consistent with previous reports (31, 34, 35) (Fig. S9A). Intriguingly, MMI treatment rendered the expression of ChREBPβ, a potent ChREBP isoform responsible for transcriptional activity 20-fold higher than that of ChREBPα (36), almost undetectable in scWAT from both f/f and KO mice (Fig. 5E). Accordingly, the differences in DNL gene expressions (*Fasn*, *Acly*) were less marked under this condition (Fig. 5F). T3 injection induced ChREBP and DNL gene expressions not only in f/f mice but also in KO mice, though apparently to a lesser extent, suggesting diminished thyroid hormone action in KO mice (Fig. 5E and F). As for PPARγ target genes, however, *Adipoq* and *Plin1* mRNA levels were decreased in scWAT from AiTFG KO mice when compared with the control even under hypothyroid conditions, suggesting a contribution(s) of mechanism(s) other than altered thyroid hormone action to the down-regulation of PPARγ target genes (Fig. 5G).

Of note, in SVF-derived adipocytes, the addition of T3 or GC-1, selective thyroid hormone receptor-beta (TRβ) agonists, increased ChREBP as well as *Fasn* mRNA expressions, but the induction of these genes was not impaired even in TFG-deleted adipocytes (Fig. S9B and C). T3 or GC-1 also induced *Ucp1* mRNA, but in contrast to the phenotype in vivo, this was paradoxically augmented in TFG-deleted adipocytes (Fig. S9D). While mRNA expression levels of thyroid hormone receptors, *Thra* and *Thrb*, were down-regulated in scWAT from AiTFG KO mice (Fig. 5H), this was not observed in TFG-deleted adipocytes (Fig. S9E).

Intriguingly, under hypothyroid conditions, Ucp1 induction in scWAT by CL-316,243 injection was markedly impaired in both f/f and KO mice, and, furthermore, there was no difference between the genotypes (Fig. 5I). It is worth noting that *Adrb3* mRNA levels differed minimally between the genotypes even with MMI treatment (Fig. S9F). These results suggest that diminished Ucp1 induction in response to adrenergic stimulation in scWAT from AiTFG KO mice (Fig. 4A and C) was due to diminished potentiation of adrenergic signaling by thyroid hormone (28), rather than direct impairment of adrenergic signaling itself (Fig. 5J).

Collectively, TFG deletion impairs thyroid hormone action in scWAT only in vivo, thereby leading to the down-regulation of adrenergic Ucp1 induction or DNL gene expressions in scWAT from AiTFG KO mice under physiological euthyroid conditions.

### Deletion of adipocyte TFG in early phase of HFD feeding impairs adipose expansion in epiWAT, whereas deletion of the TFG in hypertrophic adipocytes triggers adipocyte cell death

**Fig. 6.**
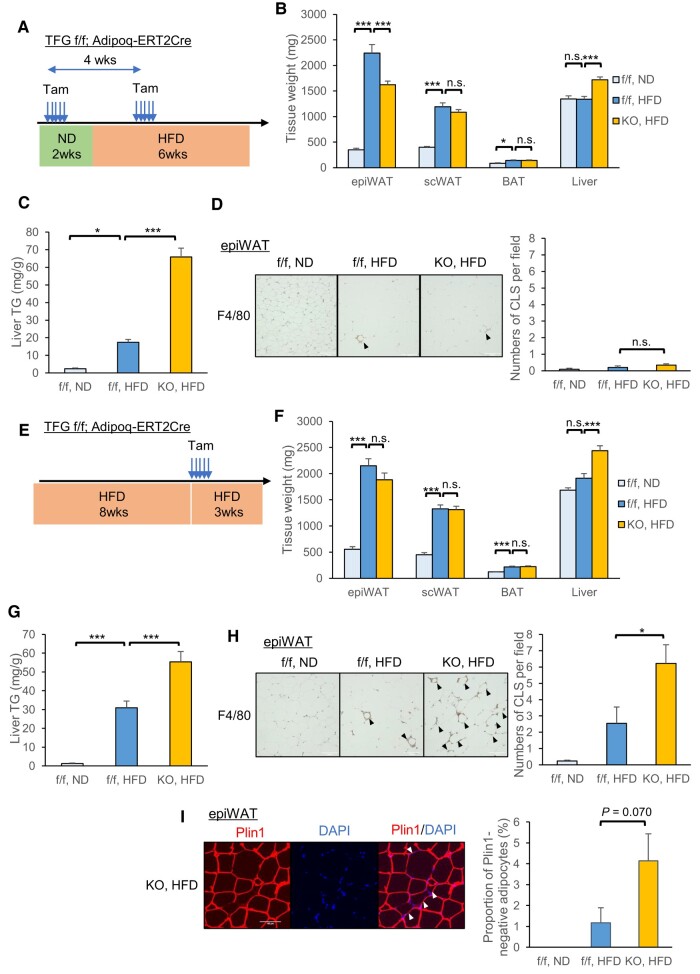
The TFG is essential for adipose expansion and for survival of hypertrophic adipocytes. A–D) Mice were injected with tamoxifen and then fed the HFD for 6 weeks. Each received a second tamoxifen injection after 2 weeks of HFD feeding to assure TFG deletion in newly differentiating as well as mature adipocytes. A) The time course of the experiment. B) Tissue weight. C) Liver TG content. (ND: *n* = 10, HFD: *n* = 22–23). D) F4/80 staining in epiWAT (arrowhead: CLS; left panel) and the quantification of CLSs (*n* = 7–9; right panel). E–I) Mice were fed the HFD for 8 weeks, received tamoxifen injections, and were then sacrificed after 3 additional weeks of HFD feeding. E) The time course of the experiment. F) Tissue weight. G) Liver TG content (ND: *n* = 13, HFD: *n* = 17–19). H) F4/80 staining in epiWAT (arrowhead: CLS; left panel) and the quantification of CLSs (*n* = 5–10; right panel). I) Plin1 staining in epiWAT from a HFD-fed KO mouse (left panel). Nuclei were stained with DAPI. The arrowheads indicate Plin1-negative (dead) adipocytes. Percentages of Plin1-negative adipocytes are shown in the right panel. Scale bar: 100 μm. *P < 0.05, ***P < 0.001.

A previous study demonstrated that in male mice, HFD feeding transiently induces proliferation of adipocyte precursor cells in the first week of this feeding, specifically in epiWAT but not in scWAT, and that mature adipocytes originating from the proliferated precursor cells are detectable when tissues are analyzed after 8 weeks of HFD feeding, indicating a contribution to adipose hyperplasia (27). Therefore, we explored the phenotypes developing in response to HFD feeding in two experimental settings: first, the deletion of the TFG prior to and shortly after starting HFD feeding (Fig. 6A), and second, deleting the TFG after 8 weeks of HFD feeding when proliferated precursor cells presumably had differentiated into mature adipocytes (Fig. 6E).

In the first experiment, tamoxifen was administered to delete the TFG in mature adipocytes 2 weeks before HFD feeding. Additional tamoxifen was injected after 2 weeks of HFD feeding to delete the TFG in newly differentiating adipocytes (Fig. 6A). After 6 weeks of HFD feeding, expansion of epiWAT tissue, but not that of scWAT, was significantly impaired in KO mice as compared to the controls (Fig. 6B), with body weights being similar for these two genotypes (Fig. S10A). HFD-fed KO mice accumulated more lipid in the liver, as evidenced by increased hepatic weight (Fig. 6B), increased TG content (Fig. 6C), and histologically apparent steatosis (Fig. S10B). Consequently, HFD-fed KO mice were more glucose intolerant (Fig. S10C) and insulin resistant (Fig. S10D). Although lipid droplets (which are nearly equal in size to adipocytes in WAT) in epiWAT were significantly smaller in HFD-fed KO mice than in HFD-fed f/f mice (Fig. S10E), the difference was less marked than the difference in epiWAT volume (Fig. 6B), which suggests that the impaired epiWAT expansion observed in HFD-fed KO mice was more likely due to impaired hyperplasia rather than impaired adipocyte hypertrophy. Notably, in this experiment, adipocyte death in epiWAT, observed as a crown-like structure (CLS) with F4/80 immunostaining, was not significantly increased in either f/f or KO mice by HFD feeding (Fig. 6D). Furthermore, down-regulation of the PPARγ target and DNL genes by adipocyte TFG deletion was apparently absent in both epiWAT (Fig. S11A) and scWAT (Fig. S11C) under HFD-fed conditions. Of note, ChREBPβ expressions in WAT were dramatically decreased, and the differences between the genotypes were no longer detectable under HFD-fed conditions (Fig. S11B and D), which might explain the diminished differences in DNL gene expressions (Fig. S11A and C).

In the second experiment, tamoxifen was administered after 8 weeks of HFD feeding, and the mice were then subjected to the experiments after an additional 3 weeks of HFD feeding (Fig. 6E). While body weights and epiWAT volumes were similar in HFD-fed f/f and KO mice (Figs. S10F and 6F), liver weight was significantly greater (Fig. 6F), and hepatic steatosis was consistently more prominent in HFD-fed KO than in HFD-fed f/f mice (Fig. 6G), leading to higher blood glucose levels in the former (Fig. S10G). In this experimental setting, TFG deletion apparently increased adipocyte death in epiWAT, as evidenced by significantly increased CLS with F4/80 staining (Fig. 6H) and by the trend favoring an increase in Plin1-negative adipocytes (Fig. 6I) as well as a trend toward increased expressions of certain inflammatory cytokines (*Tnf* and *Il6*; Fig. S10H).

These findings suggest that adipocyte TFG, the expression of which increases in response to HFD feeding, especially in epiWAT, contributes to adipogenesis and adipose tissue expansion in the early phase of HFD feeding, and is also crucial for the survival of hypertrophic adipocytes.

## Discussion

In this study, we demonstrated that TFG expression rises in response to HFD feeding, especially in epiWAT, and that the TFG is essential for adipogenesis in SVF-derived adipocytes. TFG deletion in the early phase of HFD feeding impaired adipose expansion in epiWAT, whereas TFG deletion after adipose tissue expansion triggered adipocyte death, both of which exacerbated hepatic steatosis and glucose intolerance.

Adipose tissue expansion is comprised of both hyperplasia and hypertrophy, the former of which has been proven to occur predominantly in epiWAT in male mice (27). We found (i) that the TFG is increased, especially in epiWAT with HFD feeding, and (ii) that this increase was observed not only in adipocytes but also in SVF which includes adipocyte precursor cells. Furthermore, (iii) TFG expression was found to be increased during the course of adipocyte differentiation (Figs. 2C, S3A, and S5C) and (iv) deletion of the TFG in SVF markedly impaired adipocyte differentiation. Given that (v) adipocyte cell size was not strikingly reduced by TFG deletion, impaired expansion of adipose tissue in epiWAT was deemed more likely to have been caused by impaired hyperplasia rather than impaired hypertrophy. Our observation that TFG mRNA levels correlate with body weight gain during a 1-week period of HFD feeding only in epiWAT (Fig. S2D) might also reflect its significance in adipogenesis in response to excessive nutritional intake.

The reason for TFG deletion in hypertrophic adipocytes increasing cell death has yet to be determined, but we strongly suspect that when adipocytes expand in size, an increase in cellular TFG abundance is needed to accommodate and to sustain adipocyte viability, given (i) that the TFG is essential for intracellular vesicle transport the need for which would presumably increase especially as cells become enlarged (15–17), (ii) that *tfg-1*, the TFG ortholog in *Caenorhabditis elegans*, reportedly regulates cell size (37), and (iii) that tissue-specific TFG deletion in mice increased cell death in motor neurons (24), which possess long axons, but not in pancreatic β cells (20).

Microarray analysis and real-time PCR revealed down-regulation of PPARγ target, DNL, and mitochondria-related genes in scWAT from AiTFG KO mice. Interestingly, however, these differences were mostly absent according to the analysis of TFG-deleted SVF-derived mature adipocytes, suggesting the existence of cell nonautonomous factor(s). We concluded that impaired thyroid hormone action is one of the possibly many underlying mechanisms, based on the findings (i) that Ucp1 induction by thyroid hormone was blunted in scWAT from AiTFG KO mice and (ii) that mRNA levels of thyroid hormone receptors as well as ChREBPβ, which was induced by and reportedly mediates the metabolic actions of thyroid hormone (31–33) were dramatically down-regulated in scWAT from AiTFG KO mice, without concomitant impairment of insulin signaling or insulin-stimulated glucose uptake, and (iii) that down-regulation of DNL genes in AiTFG KO mice was dependent mainly on ChREBP and became obscured when there was no difference in ChREBP expression levels between f/f and KO, such as under MMI-treated (Fig. 5F) or HFD-fed conditions (Fig. S11C). Although Ucp1 induction by adrenergic stimulation was also blunted in scWAT from AiTFG KO mice, (iv) this was likewise nullified under the MMI-treated condition, indicating impairment of adrenergic Ucp1 induction in scWAT to also be dependent on thyroid hormone action (28), which reportedly potentiates cAMP production in response to adrenergic stimulation (38, 39).

Although these findings collectively support our conclusion that TFG deletion in adipocytes impairs thyroid hormone action at least in scWAT in vivo, the response to T3 or to GC-1 in TFG-deleted SVF-derived adipocytes was not diminished, with no decrease in mRNA levels of thyroid hormone receptors. Furthermore, in BAT, despite the TFG being efficiently deleted by tamoxifen injections, TFG deletion only mildly diminished the PPARγ target gene *Adipoq* (Fig. S4E) as well as the basal and adrenergic inductions of UCP1 (Fig. 4A–D). Among thyroid hormone receptors, mRNA levels of TRβ, at least, were significantly increased by either T3 injection in vivo (Figs. 5H and S9A) or by adding T3 or GC-1 in vitro (Fig. S9E), raising the possibility that down-regulation of thyroid hormone receptors was a result rather than a cause of diminished thyroid hormone action. If so, TFG deletion presumably impairs thyroid hormone action in scWAT while having little impact in BAT, where TFG deletion slightly decreased mRNA levels of TRα but did not change either basal or T3-induced mRNA levels of TRβ (Fig. S9A). This might explain why TFG deletion attenuated PKA activation in response to CL-316,243 injection in scWAT but not in BAT (Fig. S7B and C). Further studies are needed to elucidate how TFG deletion diminished thyroid hormone action predominantly in scWAT in vivo.

Although impaired PPARγ target gene expressions could potentially be explained by impaired thyroid hormone action, since down-regulation of ChREBP and DNL limits the production of endogenous PPARγ ligands (40), the down-regulation of PPARγ target genes in AiTFG KO mice would thus persist under MMI-treated conditions (Fig. 5G). This suggests the existence of in vivo specific and thyroid hormone–independent mechanisms, which also merit further investigation.

Interestingly, TFG expression levels were significantly decreased in BAT from ob/ob mice compared with the control (Fig. S1A), possibly contributing to impaired thermogenesis in extreme obesity, since TFG deletion in BAT mildly decreased UCP1 expressions, although the mechanism underlying these observations remains unclear. Since the UCP1 promoter possesses PPAR response elements as well as cAMP response elements and thyroid hormone response elements (41), down-regulation of UCP1 might be caused by a mild attenuation of PPARγ activities in BAT, as reflected by a slight decrease in *Adipoq* mRNA.

An important limitation of our study is the use of the tamoxifen-inducible KO model. We adopted this model since pups generated by crossing TFG f/f mice with aP2-Cre transgenic mice only survived a few weeks, if they were even born, which suggested the essential roles of the TFG in adipogenesis. Although the tamoxifen-inducible KO model was useful for clarifying the role of the TFG at different time points, i.e. in early or late stages of HFD feeding, a previous study showed that tamoxifen treatment itself triggers acute adipocyte death in WAT, which is notably more predominant in epiWAT than in scWAT (42). Therefore, the increased adipocyte death observed with TFG deletion at the late stages of HFD feeding might result not solely from TFG deletion but rather from being combined with the toxicity of tamoxifen. The earlier study showed that oral tamoxifen treatment for 5 consecutive days (from days 1 to 5) decreased epiWAT and scWAT volumes by 75 and 30%, respectively, and that recovery was achieved by rapid adipogenesis with the difference having become insignificant, at least in scWAT, by day 15 (42). In our experiments, the efficiency of TFG deletion and the subsequent phenotypes were apparently less marked in epiWAT than those in scWAT (Fig. S4E–G), which might be partially due to adipocyte death and subsequent regeneration shortly after tamoxifen injections which occurred predominantly in epiWAT, taking into consideration that newly generated adipocytes might have been spared tamoxifen-induced deletion of the TFG. An alternative method(s), such as doxycycline inducible systems, might be applicable in future studies to circumvent this shortcoming.

In summary, the TFG is an essential regulator of adipose expansion in response to excessive nutritional intake, which functions by regulating adipogenesis and the survival of hypertrophic adipocytes. The TFG also regulates thyroid hormone action in scWAT, which contributes to DNL and thermogenic gene expression in scWAT. TFG deletion in adipocytes also impairs PPARγ target gene expressions and increases liver TGs (4), which is at least partly independent of its effects on thyroid hormone actions. Further studies are needed to clarify the precise molecular mechanisms by which the TFG regulates thyroid hormone actions and PPARγ activities, specifically in vivo, and its therapeutic potential for preventing steatosis and the associated insulin resistance.

## Supplementary Material

pgae150_Supplementary_Data

## Data Availability

Microarray data have been deposited with Gene Expression Omnibus (GSE143967).
