# Supplementary material for: Trk-fused gene plays a critical role in diet-induced adipose tissue expansion and is also involved in thyroid hormone action
Source: PNAS Nexus. 2024 Apr 9;3(4):pgae150. doi: 10.1093/pnasnexus/pgae150 (PMC11046318; doi:10.1093/pnasnexus/pgae150)
Supplement: pgae150_Supplementary_Data [file pgae150_supplementary_data.pdf]

Supplementary Information

Trk-fused gene (TFG) plays a critical role in diet-induced adipose tissue expansion and is also involved in thyroid hormone action

Takeshi Yamamotoya, Yukino Ohata, Yasuyuki Akasaka, Shun Hasei, Masa-Ki Inoue, Yusuke Nakatsu, Machi Kanna, Hiroki Yamazaki, Akifumi Kushiyama, Midori Fujishiro, Hiraku Ono, Hideyuki Sakoda, Tetsuya Yamada, Hisamitsu Ishihara, Tomoichiro Asano

Supplementary Material and Methods

Microarray Analysis

Total RNA was isolated using NucleoSpin RNA (Takara Bio, Kusatsu, Japan) from subcutaneous inguinal white adipose tissue (WAT) of AiTFG KO and control mice. After confirming the quality of the extracted RNA using a Bioanalyzer 2100 (Agilent Technologies), 10 ng of RNA were subjected to reverse transcription using a GeneChip WT pico Reagent Kit (Thermo Fisher Scientific) and hybridization onto a Clariom D Array, Mouse (Thermo Fisher Scientific). The arrays were scanned using the Affymetrix GeneChip Scanner 3000 7G (Affymetrix, Santa Clara, CA, USA). The data obtained were analyzed using a Transcriptome Analysis Console ver.4.0. In the pathway analysis, genes of which the expression levels differed more than 4-fold between AiTFG KO and the control mice with statistical significance ($P < 0.05$) were analyzed.

Intraperitoneal glucose tolerance test (IPGTT), insulin tolerance test (ITT), pyruvate tolerance test (PTT)

For IPGTT, mice were fasted for 17 h and then received an intraperitoneal glucose injection (2 g/kg for normal diet (ND)-fed mice and 1 g/kg for HFD-fed (and control ND-fed) mice). For ITT, mice were fasted for 4 h and then given an intraperitoneal insulin injection (0.75 unit/kg). For PTT, mice were fasted for 18 h and then received an intraperitoneal sodium pyruvate injection (2 g/kg).

2-deoxyglucose (2-DG) Uptake Assay

The experimental animals were fasted for 18 h and then given an intraperitoneal injection of insulin (0.75 unit/kg) or vehicle. A mixed solution of 10% glucose and 10% 2-DG (glucose: 2-DG ratio = 4: 1) was prepared and then injected intraperitoneally 15 min after the insulin (or vehicle) injection. The mice were sacrificed 30 min after the glucose mixture injection and tissues were immediately harvested and frozen in liquid nitrogen. Subsequently, tissue samples were lysed by sonication in 0.08 M HCl buffer. Intracellular 2-DG-6-phosphate levels were measured using a Glucose Uptake-Glo Assay (Promega) according to the manufacturer’s instructions.

Supplementary Figure legends

Supplementary Figure S1. Related to Figure 1.

(A) TFG protein levels in BAT from wild type (+/+) and ob/ob mice (n = 8).

(B) TFG protein levels in BAT from C57BL6/J male mice fed HFD for 0, 1, 4 and 8 weeks.

(**: $P < 0.01$)

Supplementary Figure S2. TFG expression levels after short-term HFD-feeding in WAT from different strains of male mice and their correlations with body weight (BW), BW gain and WAT volume.

Four strains of 7-week-old male mice (A/J, BALB/c, C57BL6/J and AKR) were subjected to 1 week of HFD feeding and TFG expression levels (protein, mRNA) in epiWAT and scWAT were then determined.

(A) TFG protein levels. (B) WAT volumes. (C) Correlations between TFG protein levels and BW, BW gain (g) and WAT volumes (n = 16). (D) Correlations between TFG mRNA levels and BW, BW gain (g) and WAT volumes (n = 20). (*: $P < 0.05$, **: $P < 0.01$, ***: $P < 0.001$)

Supplementary Figure S3. Related to Figure 2.

SVF cells were induced to undergo adipocyte differentiation on Day 0 and were then infected with AAV-EGFP or AAV-Cre on Day 1.

(A) Western blot analyses on Days 0, 1, 2, 3, 5 and 8.

(B) mRNA levels on Day 8 (n = 4).

(*: $P < 0.05$, ***: $P < 0.001$)

Supplementary Figure S4. Related to Figure 3.

(A) Tissue weight normalized by body weight (BW) (n = 8-9)

(B) Intraperitoneal glucose tolerance test (Glucose 2 g/kg i.p.) (n = 6).

(C) Insulin tolerance test (Insulin 0.75 U/kg i.p.) (n = 5-6)

(D) Pyruvate tolerance test (Sodium pyruvate 2 g/kg i.p.) (n = 7)

(E-G) mRNA levels of *Tfg*, *Pparg* and its target genes (E), DNL genes (F) and genes related to mitochondrial function and thermogenesis (G) in epiWAT (left panels) and BAT (right panels) (n = 8). (*: $P < 0.05$, **: $P < 0.01$, ***: $P < 0.001$)

Supplementary Figure S5. TFG deletion in differentiated SVF-derived adipocytes did not downregulate PPARγ target, DNL or mitochondria-related gene expressions.

(A) SVF isolated from scWAT of TFG f/f mice were differentiated into mature adipocytes, infected with AAV-EGFP (control) or AAV-Cre on Day -4 and analysis was conducted on Day 12.

(B) Representative microscopic images (scale bar: 100 μm). (C) Western blot analysis.
(D-F) mRNA levels of *Tfg*, *Pparg* and its target genes (D), DNL genes (E) and genes related to mitochondrial function and thermogenesis (F) (n = 4).
(*: $P < 0.05$, **: $P < 0.01$, ***: $P < 0.001$)

Supplementary Figure S6. Browning of scWAT by CL-316,243 injection was impaired in AiTFG KO.
(A) The time-course of the experiment.
(B) *Ucp1* mRNA levels in scWAT (n = 5-8).
(C) UCP1 immunostaining in scWAT (scale bar: 100 μm).
(*: $P < 0.05$)

Supplementary Figure S7. β-adrenergic receptor-mediated PKA activation is slightly impaired in scWAT from AiTFG KO.
(A) mRNA levels of Adrb3 in scWAT and BAT (n = 8)
(B, C) Mice were injected with CL-316,243 (1 mg/kg) or vehicle and then sacrificed 1 h later. The levels of phospho-PKA substrate and phosphorylations of HSL at Ser563 and Ser660 in scWAT (B) and BAT (C) were analyzed by Western blotting.
(*: $P < 0.05$)

Supplementary Figure S8. ChREBP mRNA levels are downregulated in scWAT from AiTFG KO mice.
(A) mRNA levels of ChREBP (*Mlxipl*) (total, alpha and beta isoforms) in scWAT (left panel), epiWAT (middle panel) and BAT (right panel) (n = 8).
(B) Insulin-induced phosphorylation of Akt (Ser473) in epiWAT, scWAT, liver and muscle. Mice were injected with insulin (0.75 U/kg) or vehicle and then sacrificed 15 min later.
(C) Glucose uptake assay *in vivo*. Mice were injected with insulin (0.75 U/kg) or vehicle and were subsequently administered a mixture of glucose and 2-DG before being sacrificed. Intracellular 2-DG-6-phosphate levels in epiWAT and scWAT were measured using a Glucose Uptake-Glo Assay 2DG6P (n = 3-5).
(D) mRNA levels of thyroid hormone receptors (*Thra*, *Thrb*) and iodothyronine deiodinase 2 (*Dio2*) in scWAT (n = 8).
(*: $P < 0.05$, **: $P < 0.01$, ***: $P < 0.001$)

Supplementary Figure S9. Related to Figure 5.
(A) mRNA levels of *Tfg*, *Ucp1* and thyroid hormone receptors (*Thra, Thrb*) in BAT (n = 7-9).

(B-E) SVF isolated from scWAT of TFG f/f mice were differentiated into mature adipocytes and infected with AAV-EGFP or AAV-Cre on Day 4. The medium for incubation was switched to high-glucose medium (25 mM) with charcoal-stripped serum, since ChREBPβ mRNA was almost undetectable when cultured in low-glucose medium (5.5 mM). Cells were harvested on Day 12 after incubation with T3 (100 nM), GC-1 (100 nM) or vehicle for the final 6 days. mRNA levels of *Tfg* and DNL genes (B), ChREBP (*Mlxipl*) (C), *Ucp1* (D) and thyroid hormone receptors (E) (n = 3-4).
(F) mRNA levels of *Adrb3* in scWAT (n = 7-9).
(*: $P < 0.05$, **: $P < 0.01$, ***: $P < 0.001$)

Supplementary Figure S10. Related to Figure 6.
(A-E) Related to the experiment shown in Fig.6A-D.
(A) Body weight (ND: n = 10, HFD: n = 22-23). (B) H-E staining of the liver from HFD-fed mice (scale bar: 100 μm).
(C-D) Intraperitoneal glucose tolerance (Glucose 1 g/kg i.p.) (C) and insulin tolerance (Insulin 0.75 U/kg i.p.) tests (D) performed when the mice were fed HFD for 5 weeks (n = 5-7).
(E) Lipid droplet size (left panel) and frequency (right panel) in epiWAT. Sections were stained with Plin1 and the areas were measured using Image J.
(F-H) Related to the experiment shown in Fig.6E-I.
(F) Body weight (G) Blood glucose levels (fed ad libitum) (ND: n = 13, HFD: n = 17-19)
(H) mRNA levels of *Tfg* and inflammatory cytokines (n =4-10).
(*: $P < 0.05$, **: $P < 0.01$, ***: $P < 0.001$)

Supplementary Figure S11. Downregulation of DNL genes by TFG deletion was not observed in HFD-fed settings, in which ChREBPβ expression was markedly decreased.
(A-D) Related to the experiment shown in Fig.7A-D.
(A, C) mRNA levels of *Tfg*, *Pparg* and its target genes and DNL genes in epiWAT (A) and scWAT (C) (n = 7-9).
(B, D) mRNA levels of ChREBP (*Mlxipl*) in epiWAT (B) and scWAT (D) (n = 7-9).
(**: $P < 0.01$, ***: $P < 0.001$)

Supplementary Table S1. Results from the microarray analysis of total RNA isolated from scWAT.
The results obtained from pathway analysis in the microarray analysis of total RNA isolated from scWAT. The top 13 pathways, suggested by genes of which the expression levels differed by more than 4-fold between AiTFG KO and the controls, i.e., with statistical significance ($P < 0.05$), are shown.

Supplementary Table S2. List of qPCR primers (mouse).

Supplementary Figure S1

A

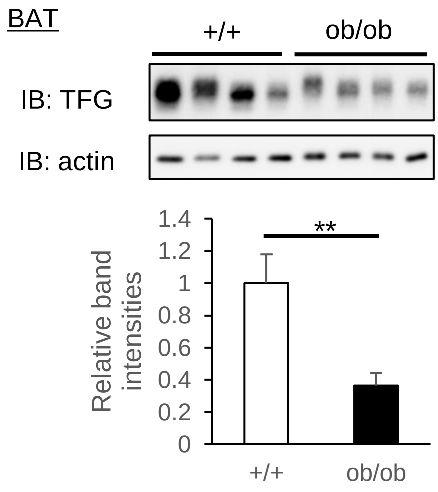

B

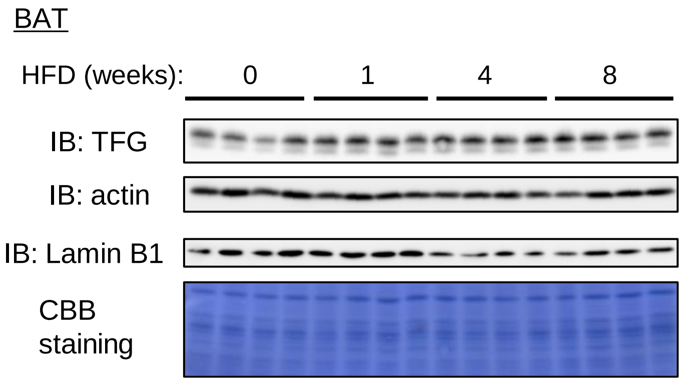

Supplementary Figure S2

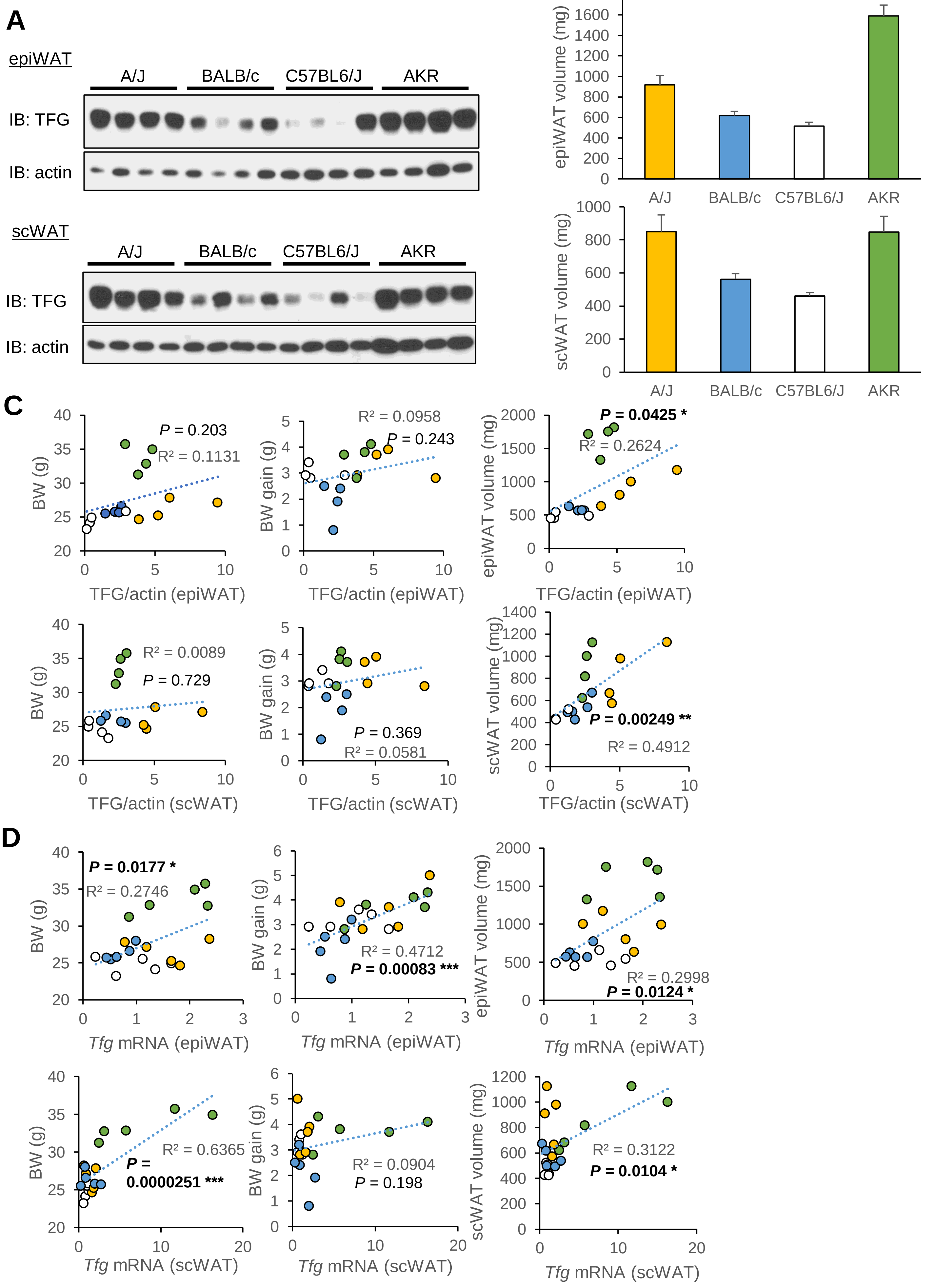

Supplementary Figure S3

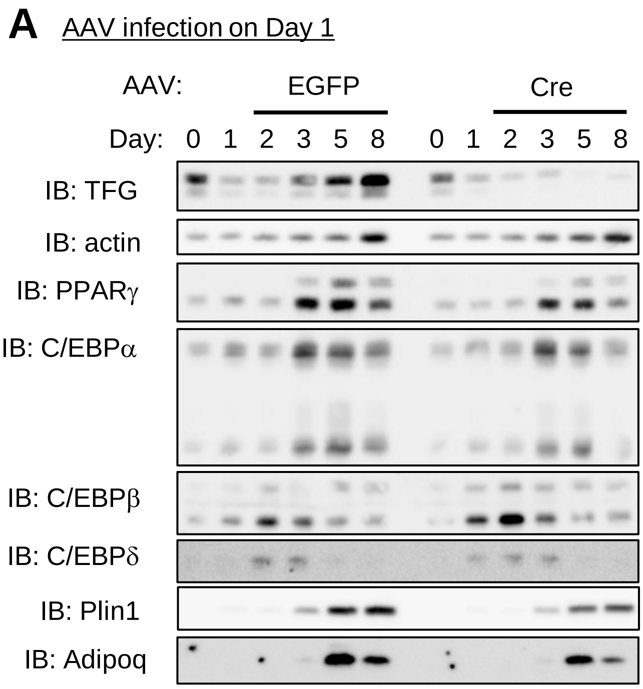

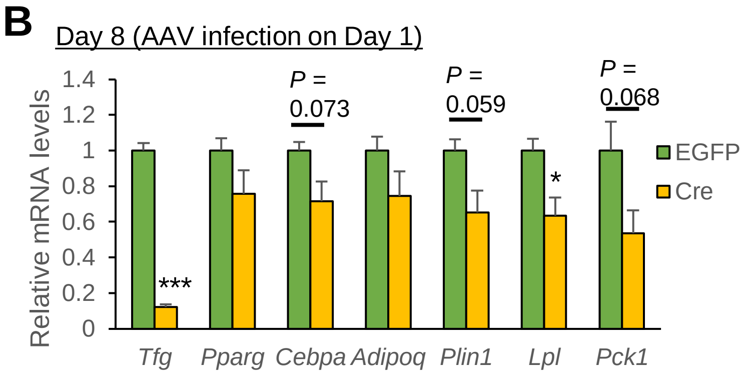

Supplementary Figure S4

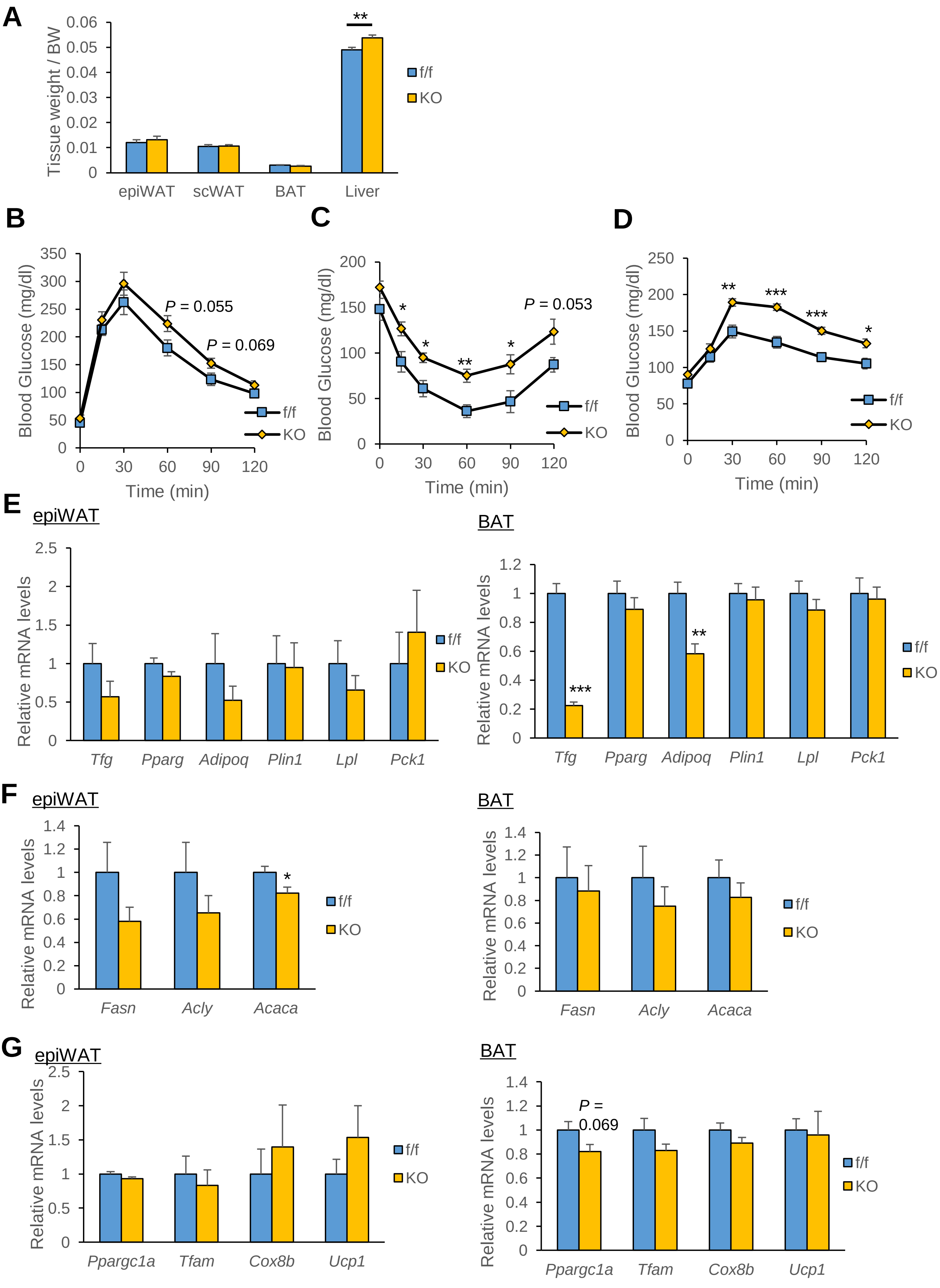

Supplementary Figure S5

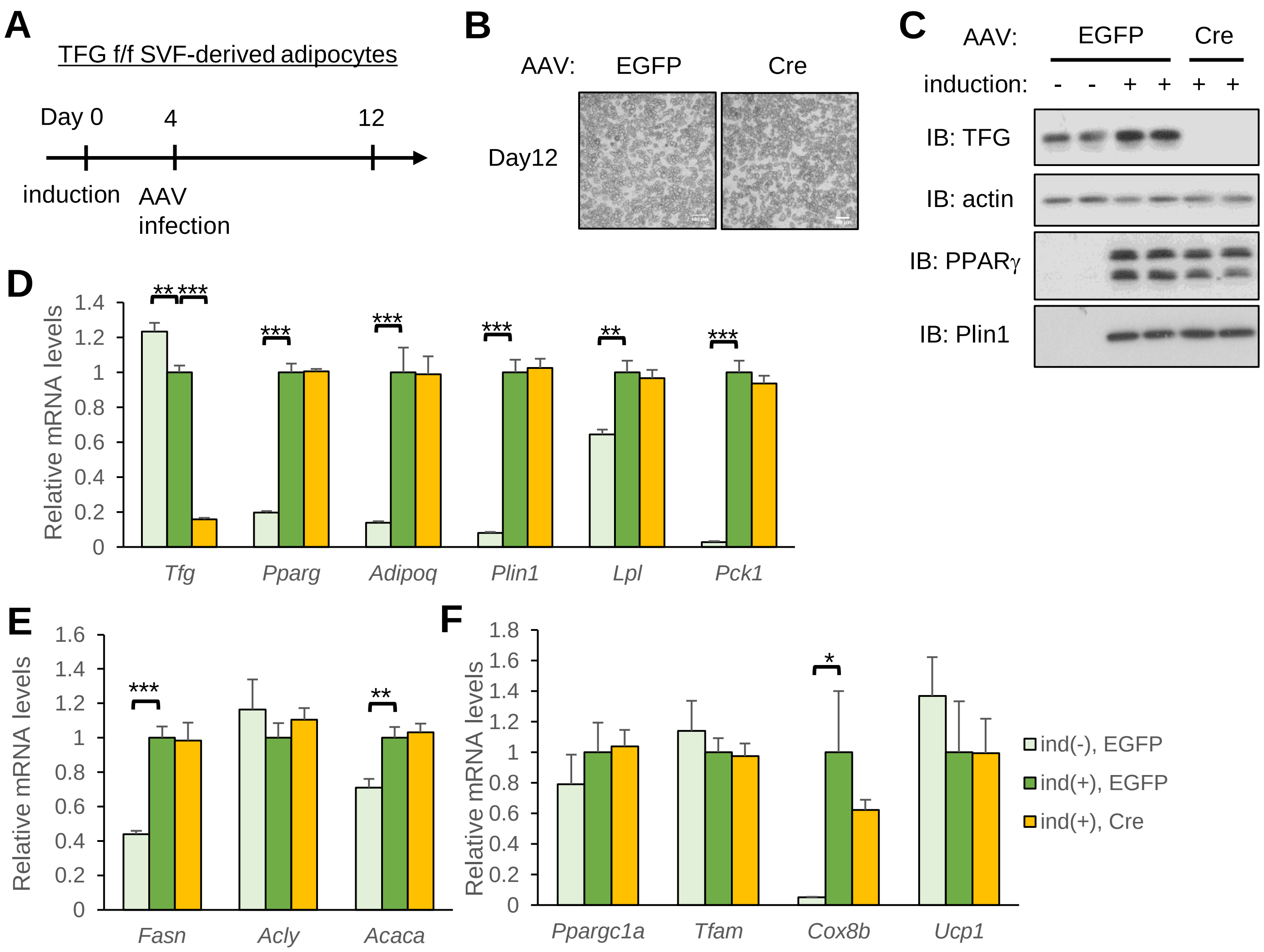

Supplementary Figure S6

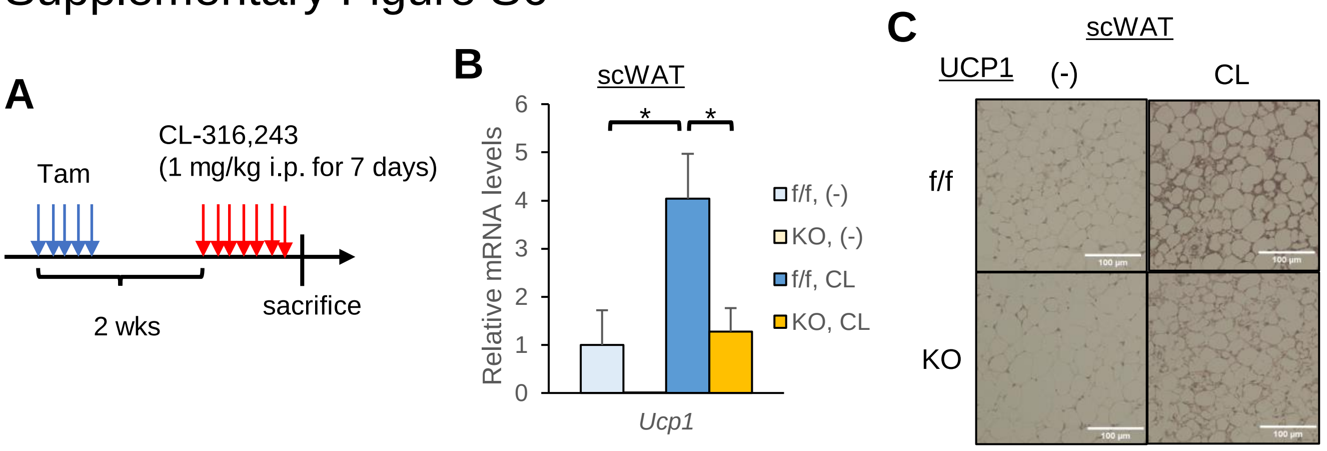

Supplementary Figure S7

A

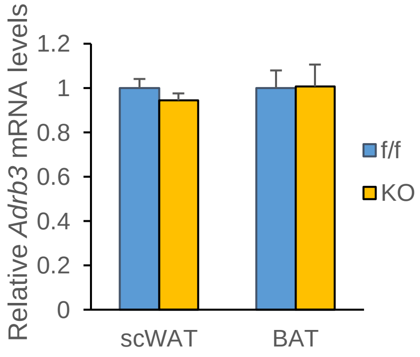

B

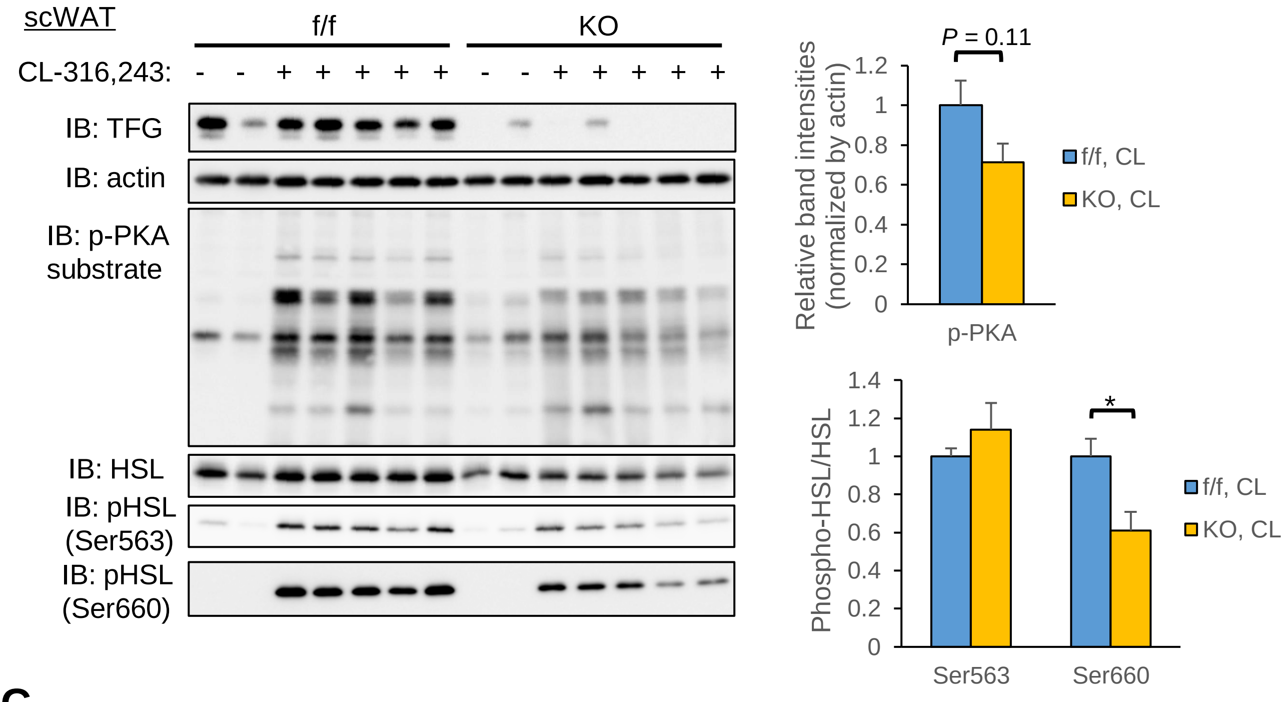

C

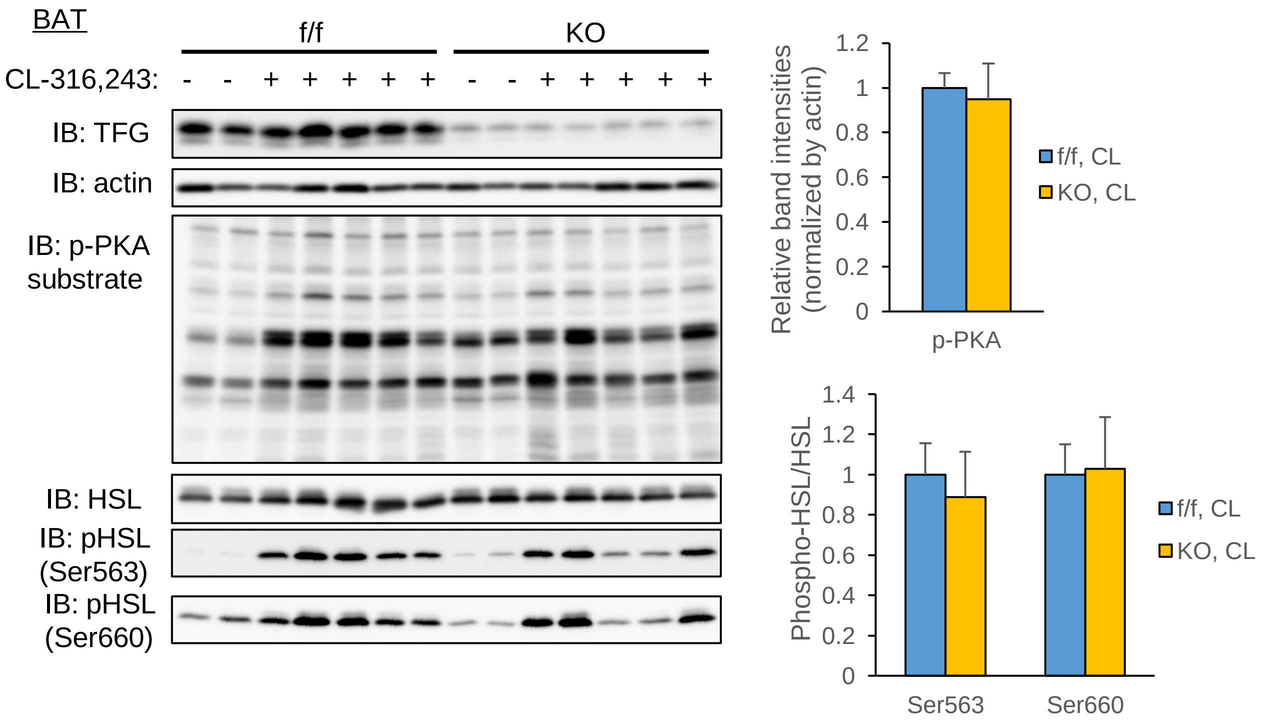

Supplementary Figure S8

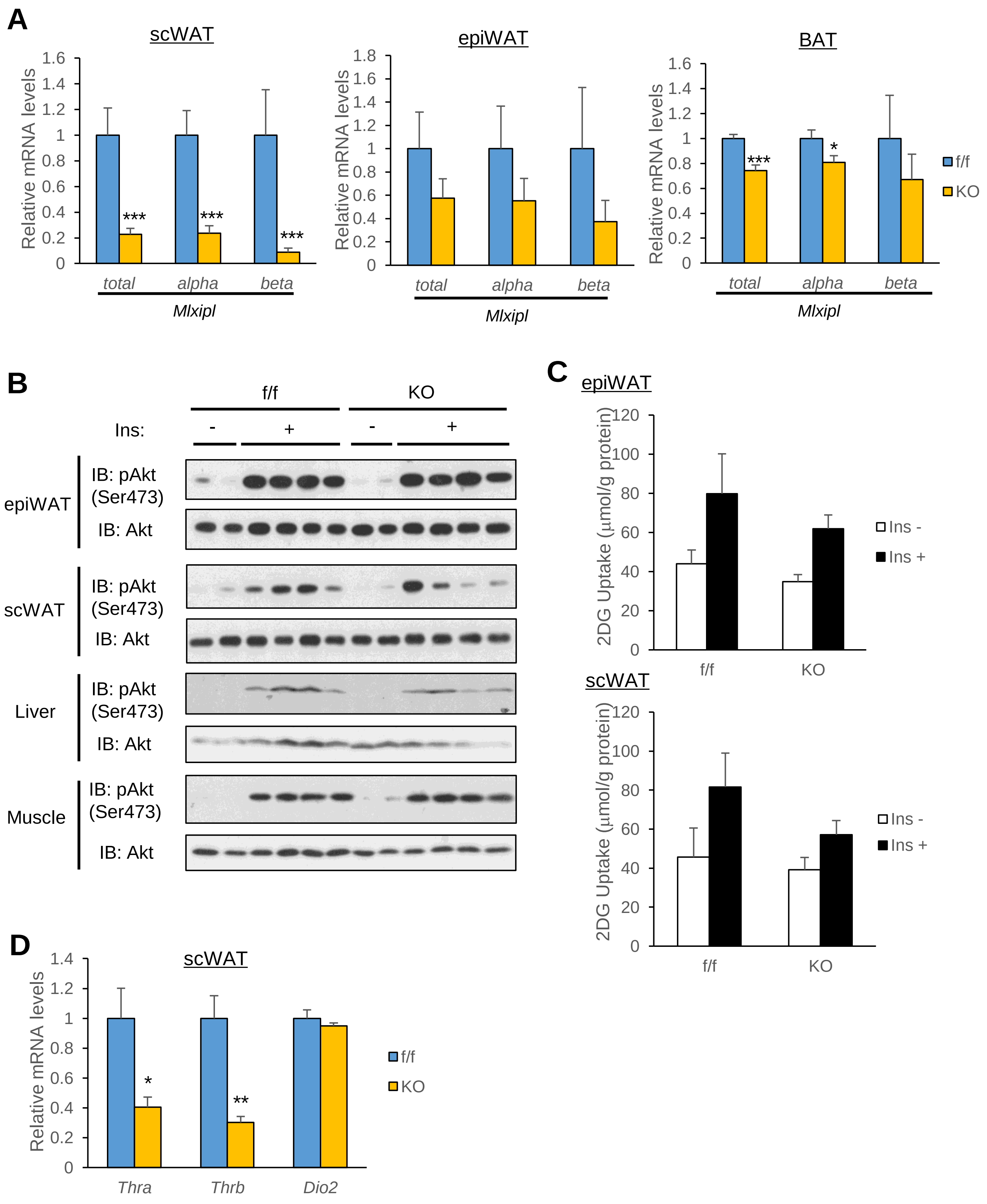

Supplementary Figure S9

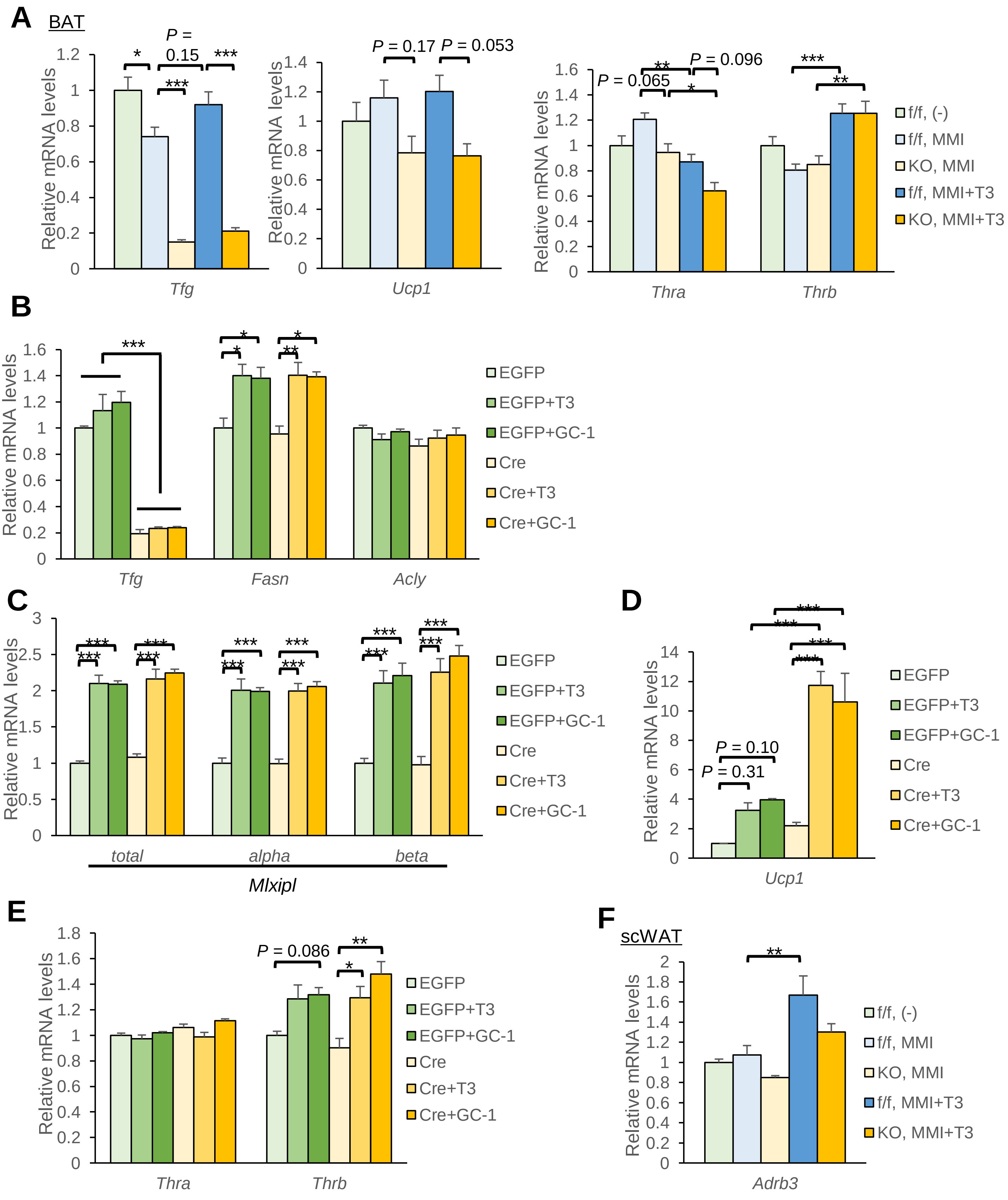

Supplementary Figure S10

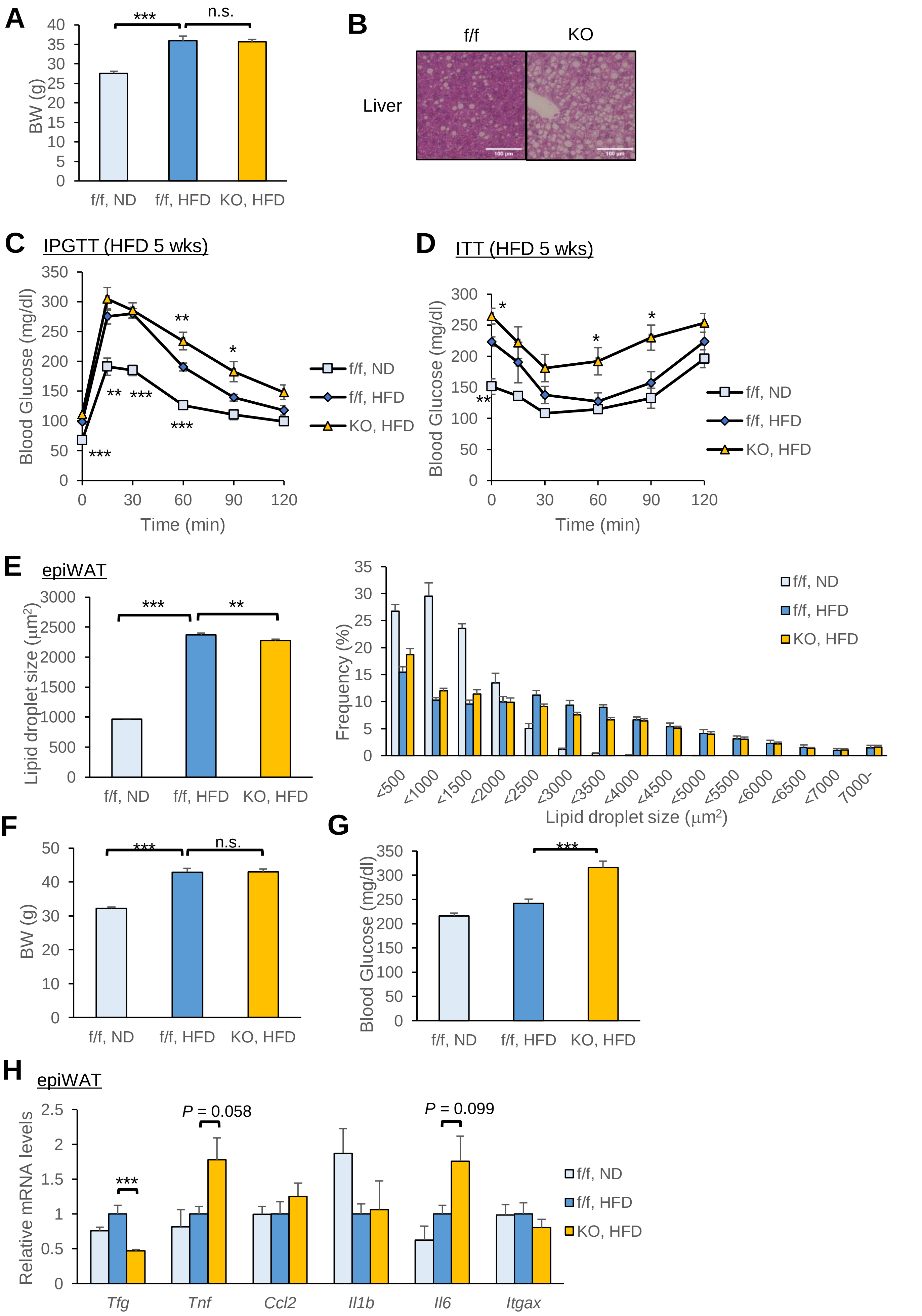

Supplementary Figure S11

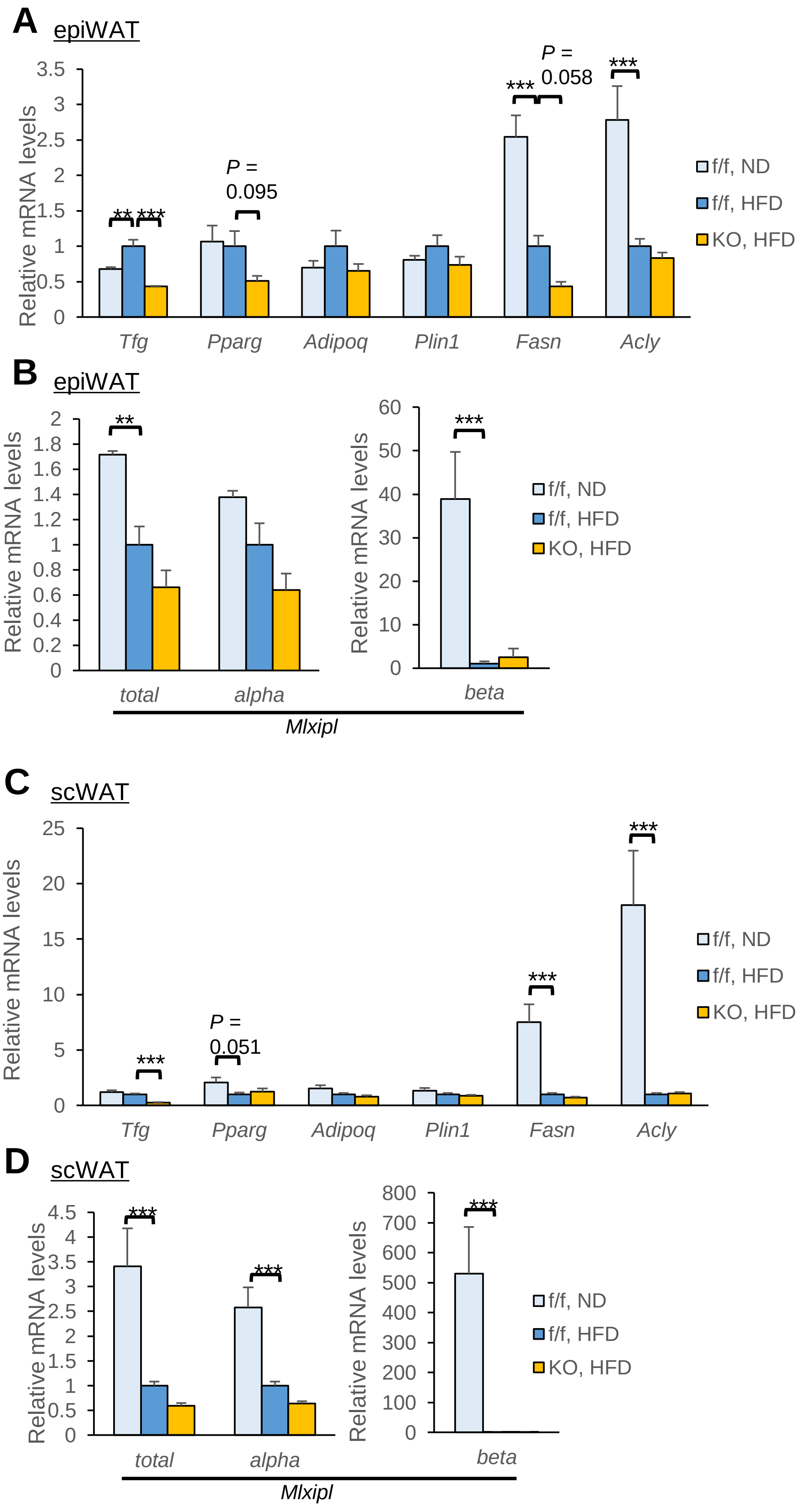

Supplementary Table S1

Pathway	#Total	#Up	Up List	#Down	Down List	Significance	p-value
Electron Transport Chain	23	0		23	Atp5a1,Cox6b1,Uqcr10,Atp5b,Ndufv2,Ndufb9,Ndufa2,Cox5a,Cox4i1,Uqcrh,Ndufa9,Ndufb3,Cox7a2,Sdha,Uqcrq,Uqcrb,Ndufb7,Ndufa1,Cox8a,Ndufc2,Ndufa4,Ndufv3,Ndufa10	21.59	0
PPAR signaling pathway	16	0		16	Pparg,Ubc,Slc27a1,Aqp7,Acox1,Acadm,Cd36,Me1,Adipoq,Plin1,Pck1,Acsl1,Scd1,Lpl,Ehhadh,Acadl	13.85	0
Oxidative phosphorylation	11	0		11	Atp5a1,Atp5b,Ndufa2,Ndufa10,Ndufa4,Ndufa9,Ndufb7,Ndufc2,Ndufb9,Ndufv2,Ndufv3	9.66	0
Fatty Acid Biosynthesis	7	0		7	Fasn,Acaca,Scd1,Acacb,Acsl1,Pcx,Acaa2	8	0
Mitochondrial LC-Fatty Acid Beta-Oxidation	6	0		6	Ehhadh,Acsl1,Hadha,Acadl,Acadm,Slc25a20	7.42	0
TCA Cycle	7	0		7	Pdhb,Pdk4,Cs,Sdha,Idh3g,Ogdh,Pcx	6.85	0
Fatty Acid Beta Oxidation (streamlined)	7	0		7	Hadha,Acadm,Acadl,Acsl1,Slc25a20,Cd36,Slc27a1	6.75	0
Fatty Acid Beta Oxidation	7	0		7	Slc25a20,Acsl1,Hadha,Acadl,Pnpla2,Lpl,Acadm	6.46	0
Retinol metabolism	7	0		7	Rbp7,Lpl,Cyp2e1,Retsat,Cd36,Adh1,Rbp4	6.13	0.000001
Arachidonate Epoxygenase Epoxide Hydrolase	3	0		3	Ephx2,Cox5a,Cox8a	5.63	0.000002
TCA Cycle (streamlined)	4	0		4	Cs,Sdha,Idh3g,Ogdh	3.99	0.000102
Triacylglyceride Synthesis	4	0		4	Gpd1,Dgat2,Lpl,Pnpla2	3.65	0.000223
Adipogenesis genes	8	0		8	Lpl,Pparg,Scd1,Epas1,Lpin1,Pck1,Retn,Adipoq	3.37	0.000424

Supplementary Table S2

	Forward	Reverse
Gapdh	TGATGGGTGTGAACCACGAG	GGGCCATCCACAGTCTTCTG
β-actin	AGGGTGTGATGGTGGGAATG	GGTTGGCCTTAGGGTTCAGG
Tfg	CCTCGGCAAGCGGGTTTTT	AGCTTTCCTCTGAATACTCGCT
Pparg	GTGGCCATCCGAATTTTTCA	CATGAATCCTTGGCCCTCTG
Cebpa	TTACAACAGGCCAGGTTTCC	CTCTGGGATGGATCGATTGT
Cebpb	GACGGTGGACAAGCTGAGCG	CCTTGTGCTGCGTCTCCAGG
Cebpd	AAAGTGCAGGCTTGTGGACT	TTACTCCACTGCCCACCTGT
Adipoq	GTCAGTGGATCTGACGACACCAA	ATGCCTGCCATCCAACCTG
Plin1	CACCATGCAAACCACAGCA	GGTCGTCATGGCTCTCATCC
Lpl	GGACGGTAACGGGAATGTATG	ACGTTGTCTAGGGGGTAGTTAAA
Pck1	CTTTGGTGGCCGTAGACCTG	AGCCAGTGGGCCAGGTATTT
Cox8b	AAGCCCATGTCTCTGCCAAG	CTTCATGCTGCGGAGCTCTT
Fasn	TTGCTGGCACTACAGAATGC	AACAGCCTCAGAGCGACAA
Acly	TTCGTCAAACAGCACTTCC	ATTTGGCTTCTTGGAGGTG
Acaca	GAGAGGGGTCAAGTCCTTCC	CTGCTGCCGTCATAAGACAA
Ppargc1a	GCACTTCGGTCATCCCTGTC	GGCGACACATCGAACAATGA
Tfam	AAGCTTCCAGGAGGCAAAGG	TGTCTCCGGATCGTTTCACA
Ucp1	ACCACCCTGGCAAAAACAGA	CCTCTGTAGGCTGCCCAATG
Mlxipl (total)	CACTCAGGGAATACACGCCTAC	ATCTTGGTCTTAGGGTCTTCAGG
Mlxipl (alpha)	CGACACTCACCCACCTCTTC	TTGTTCAGCCGGATCTTGTC
Mlxipl (beta)	TCTGCAGATCGCGTGGAG	CTTGTCCCGGCATAGCAAC
Thra	ATGGAACAGAAGCCAAGCAA	TTCCATCTGGTGACCTGGCA
Thrb	AGGGTACCACTATCGCTGCA	GCGGGTGACTTTGTCTATGA
Adrb3	ACTGCTAGCATCGAGACCTTG	AAGGGTTGGTGACAGCTAGG
